# Human fecal contamination of water, soil, and surfaces in households sharing poor-quality sanitation facilities in Maputo, Mozambique

**DOI:** 10.1016/j.ijheh.2020.113496

**Published:** 2020-05

**Authors:** David A. Holcomb, Jackie Knee, Trent Sumner, Zaida Adriano, Ellen de Bruijn, Rassul Nalá, Oliver Cumming, Joe Brown, Jill R. Stewart

**Affiliations:** a Department of Environmental Sciences and Engineering, Gillings School of Global Public Health, University of North Carolina at Chapel Hill, Chapel Hill, NC, United States; b School of Civil and Environmental Engineering, Georgia Institute of Technology, Atlanta, GA, United States; c We Consult, Maputo, Mozambique; d Departamento de Geografia, Universidade Eduardo Mondlane, Maputo, Mozambique; e Instituto Nacional de Saúde, Ministério da Saúde, Maputo, Mozambique; f Department of Disease Control, London School of Hygiene & Tropical Medicine, London, United Kingdom

**Keywords:** Microbial source tracking, Real-time quantitative PCR, Bayesian statistics, Detection limit, Water, sanitation, and hygiene (WaSH), Maputo sanitation (MapSan) trial

## Abstract

Identifying the origin of fecal contamination can support more effective interventions to interrupt enteric pathogen transmission. Microbial source tracking (MST) assays may help to identify environmental routes of pathogen transmission although these assays have performed poorly in highly contaminated domestic settings, highlighting the importance of both diagnostic validation and understanding the context-specific ecological, physical, and sociodemographic factors driving the spread of fecal contamination. We assessed fecal contamination of compounds (clusters of 2–10 households that share sanitation facilities) in low-income neighborhoods of urban Maputo, Mozambique, using a set of MST assays that were validated with animal stool and latrine sludge from study compounds. We sampled five environmental compartments involved in fecal microbe transmission and exposure: compound water source, household stored water and food preparation surfaces, and soil from the entrance to the compound latrine and the entrances to each household. Each sample was analyzed by culture for the general fecal indicator *Escherichia coli* (cEC) and by real-time PCR for the *E. coli* molecular marker EC23S857, human-associated markers HF183/BacR287 and Mnif, and GFD, an avian-associated marker. We collected 366 samples from 94 households in 58 compounds. At least one microbial target (indicator organism or marker gene) was detected in 96% of samples (353/366), with both *E. coli* targets present in the majority of samples (78%). Human targets were frequently detected in soils (59%) and occasionally in stored water (17%) but seldom in source water or on food surfaces. The avian target GFD was rarely detected in any sample type but was most common in soils (4%). To identify risk factors of fecal contamination, we estimated associations with sociodemographic, meteorological, and physical sample characteristics for each microbial target and sample type combination using Bayesian censored regression for target concentration responses and Bayesian logistic regression for target detection status. Associations with risk factors were generally weak and often differed in direction between different targets and sample types, though relationships were somewhat more consistent for physical sample characteristics. Wet soils were associated with elevated concentrations of cEC and EC23S857 and odds of detecting HF183. Water storage container characteristics that expose the contents to potential contact with hands and other objects were weakly associated with human target detection. Our results describe a setting impacted by pervasive domestic fecal contamination, including from human sources, that was largely disconnected from the observed variation in socioeconomic and sanitary conditions. This pattern suggests that in such highly contaminated settings, transformational changes to the community environment may be required before meaningful impacts on fecal contamination can be realized.

## Highlights

• Sanitation is a pervasive and understudied challenge in crowded, urban settlements.
• We applied microbial source tracking to understand routes of pathogen transmission.
• Human fecal microbes were common in soil and stored water, but not source water.
• Fecal contamination was observed regardless of modifying conditions in households.
• Transformational changes are likely needed to prevent enteric disease transmission.

## 1 Introduction

Strategies to prevent fecal-oral disease often focus on interrupting environmentally mediated transmission of fecal pathogens (Julian, 2016; Penakalapati et al., 2017; Wagner and Lanoix, 1958). The classic F-diagram conceptualizes fecal-oral transmission on separate, unidirectional pathways through each environmental reservoir, suggesting clear opportunities for intervention (Mara et al., 2010; Wagner and Lanoix, 1958). However, a single environmental reservoir may be contaminated by feces with different origins transmitted by multiple interacting pathways (Ercumen et al., 2017b; Harris et al., 2013; Schriewer et al., 2015; Vujcic et al., 2014), which can reduce the effectiveness of interventions (Briscoe, 1984). In particular, the role of animals in the spread of fecal contamination and pathogens has recently been increasingly recognized (Delahoy et al., 2018; Penakalapati et al., 2017; Prendergast et al., 2019). Several recent water, sanitation, and hygiene (WaSH) intervention trials have highlighted the need to better understand the environmental exposure and transmission dynamics driving fecal-oral disease (Cameron et al., 2013; Clasen et al., 2014; Humphrey et al., 2019; Luby et al., 2018; Null et al., 2018; Patil et al., 2014; Pickering et al., 2015; Sclar et al., 2016; Sinharoy et al., 2017).

As the rapidly urbanizing populations of many low- and middle-income countries (LMICs) increasingly reside in crowded, informal settlements that lack basic services, identifying the factors influencing fecal contamination in dense urban environments is especially relevant (Ezeh et al., 2017; Hawkins et al., 2013; McGranahan, 2015; Sinharoy et al., 2019). In many low-income urban settings, limited space and resources often require multiple households to share sanitation facilities, circumstances that may increase diarrhea risk and complicate arrangements for cleaning and maintenance (Baker et al., 2016; Heijnen et al., 2015; Shiras et al., 2018b; Simiyu et al., 2017). Such decentralized sanitation infrastructure is primarily intended to contain human excreta, but animals are also common in both public and private urban spaces and can be important sources of enteric pathogens in urban environments where pets or livestock are kept in close quarters (Baker et al., 2018; Barnes et al., 2018; Berendes et al., 2018; Harris et al., 2016; Navab-Daneshmand et al., 2018).

Fecal contamination has traditionally been evaluated by measuring fecal indicator bacteria (FIB) like *Escherichia coli* (Field and Samadpour, 2007). FIB have been consistently found at densities greater than 100 organisms per gram in domestic soils, and have been frequently observed in a variety of household settings globally (Capone et al., 2019; Ercumen et al., 2018b, 2017b; Finch et al., 1978; Pickering et al., 2012; Scott et al., 1982). However, FIB are unable to directly identify fecal sources and do not necessarily represent recent fecal contamination, as naturalized FIB have been reported in many environments (Byappanahalli and Fujioka, 2004; Field and Samadpour, 2007; Oh et al., 2012; Rivera et al., 1988; Solo-Gabriele et al., 2000). Sanitation interventions intended to interrupt domestic fecal transmission in low- and middle-income countries have not generally demonstrated an impact on ambient FIB, suggesting the interventions did not adequately address the pathways and fecal sources driving contamination in such settings (Ercumen et al., 2018a, 2018b; Sclar et al., 2016).

Molecular microbial source tracking (MST) enables fecal source identification by testing samples for the genetic material of gut microbes thought to be specific to a particular host, such as humans or ruminants (Field and Samadpour, 2007; Harwood et al., 2014). MST typically targets obligate anaerobes that strongly suggest recent fecal contamination and may provide signals with greater relevance to understanding and addressing patterns of fecal contamination (McLellan and Eren, 2014). Several studies applying MST in both rural and urban contexts have implicated livestock as a major source of domestic fecal contamination (Boehm et al., 2016; Harris et al., 2016; Schriewer et al., 2015) and human contamination was widespread among households in an urban slum (Bauza et al., 2017). While the relationships between MST markers, fecal pathogen occurrence, and health risks have yet to be well-characterized in domestic contexts, both human- and livestock-associated MST markers in rural Indian homes were associated with increased child diarrhea risk (Korajkic et al., 2018; Odagiri et al., 2016). Because MST targets the gut microbiota, which varies among populations, it is necessary to validate MST assays in each new location to determine whether the selected microbial targets are both present and unique to the intended fecal source in the study area (Stewart et al., 2013). Most MST assays were developed for water quality monitoring purposes and have often performed poorly in highly-contaminated domestic settings, reaffirming the importance of diagnostic validation (Harris et al., 2016; Odagiri et al., 2015).

This cross-sectional study investigated the sources and patterns of fecal contamination in a dense urban setting before the implementation of an onsite sanitation intervention. We validated and applied a set of molecular MST assays in households sharing poor-quality sanitation facilities in Maputo, Mozambique and assessed risk factors of fecal contamination in multiple domestic transmission pathways. In consideration of a planned intervention to contain human feces, we measured human-associated fecal microbes to identify fecal contamination that could be impacted as well as animal-associated fecal microbes less likely to be affected. Given the generally poor sanitary conditions among study households, we hypothesized that indicators of fecal contamination, including human- and animal-associated MST markers, would be detected frequently across multiple environmental compartments but would differ according to household characteristics.

## 2 Materials and methods

### 2.1 Study setting

We conducted this study in the context of the Maputo Sanitation (MapSan) trial, a controlled, before-and-after study of urban sanitation and child health (Brown et al., 2015). The majority of households in Maputo (89%) use onsite sanitation (Blackett et al., 2014), much of which fails to meet the UNICEF/WHO Joint Monitoring Programme definition of at least basic sanitation, such as a private pit latrine with a slab, to which barely half of urban Mozambicans are estimated to have access (WHO/UNICEF, 2019). Frequent flooding, high population density, and inadequate management of three-quarters of the city's fecal waste contribute to a large burden of enteric infection (86% prevalence among children under four in the MapSan cohort at baseline) and child mortality (Blackett et al., 2014; Knee et al., 2018; Sitoe et al., 2018; UN-HABITAT, 2014). The MapSan trial evaluated a latrine intervention implemented in compounds—defined household clusters sharing an outdoor courtyard—with existing sanitation facilities in poor condition and shared by the households in the compound. Intervention latrines were likewise shared by compound residents from multiple households but were separated from the wider community, typically by an existing physical barrier around the compound perimeter (Shiras et al., 2018a). Frequency-matched control compounds with similarly poor-quality shared sanitation were enrolled concurrently from the same unplanned, low-income neighborhoods of urban Maputo. In each compound, all households with children under four years of age were invited to participate. We conducted a cross-sectional baseline assessment of domestic fecal contamination at an opportunistically selected subset of study compounds from both treatment arms as they were enrolled in the MapSan pre-intervention survey during May–August 2015.

### 2.2 Ethics statement

This study was approved by the Institutional Review Board of the University of North Carolina at Chapel Hill (IRB # 15–0963). The associated MapSan trial was pre-registered at ClinicalTrials.gov (NCT02362932) and was approved by the Comité Nacional de Bioética para a Saúde (CNBS), Ministério da Saúde, Republic of Mozambique (333/CNBS/14), the Ethics Committee of The London School of Hygiene & Tropical Medicine (reference # 8345), and the Institutional Review Board of the Georgia Institute of Technology (protocol # H15160). Environmental samples were collected only from households with children under four years old enrolled in the MapSan study, for whom a parent or guardian had provided written informed consent. Verbal assent was obtained from the head of each compound prior to initiating enrollment and sampling activities.

### 2.3 Sample collection

We sampled three potential environmental reservoirs of fecal contamination—water, soil, and surfaces—at five hypothesized nodes of transmission and exposure: source water, stored water, food preparation surfaces, latrine entrance soil, and household entrance soil. Source water and latrine soil samples were collected once from each compound, while stored water, food surfaces, and household soil samples were collected from each household with children enrolled in the MapSan trial. Samples were collected by the researchers concurrent with data collection activities by the MapSan child health study field team, typically between 8:00 and 13:00 (UTC+2). Samples were immediately placed on ice for transport and maintained at 4 °C upon arrival at the laboratory in central Maputo. Water samples were processed within 8 h of collection; soil and surface swab samples were also usually processed the same day and always within 30 h of collection (Harmel et al., 2016; Pope et al., 2003).

Water samples were collected in approximately 1 L volumes in sterile plastic sample bags and immediately treated with approximately 20 mg of sodium thiosulfate (Brim Technologies, Eatontown, NJ, USA) to neutralize residual chlorine. Source water samples were collected directly, generally from a standpipe in the compound yard. In compounds with multiple water points, a water point identified as the primary water source for one of the households with enrolled children was selected for sampling. We asked a resident of each enrolled household to provide drinking water from a storage container as if they were giving water to a child to drink. The storage container material, mouth width, presence of a lid, and water extraction method were recorded. We also asked the household respondent to provide a surface regularly used to prepare foods in the condition in which it would typically be used. A 10 cm × 10 cm template was disinfected with 10% bleach followed by 70% ethanol and placed on the surface. A sterile, flocked nylon swab (Copan Diagnostics, Murrieta, CA, USA) was wetted in a centrifuge tube containing 12 mL sterile ¼-strength Ringer's solution (Oxoid, Hampshire, UK) and swabbed within the template in 3 directions to ensure complete coverage (Hedin et al., 2010; Moore and Griffith, 2007). The swab was clipped with disinfected scissors into the centrifuge tube, and the surface was again swabbed with a second, dry swab to collect any remaining wetting solution; the second swab was likewise clipped into the same tube. On surfaces with sufficient area, this procedure was repeated on a second 100 cm^2^ area to increase the sample volume available for analysis. The surface type (e.g., table or bowl) and material were recorded.

Soil was collected 1 m in front of the compound latrine entrance and 1 m in front of the primary entrance to each household with enrolled children. A 10 cm × 10 cm square was drawn in the soil using a disinfected metal scoop. We used the scoop to gently homogenize the top 1–2 cm of soil and transfer it to a sterile sample bag (Pickering et al., 2012). Qualitative assessment of soil exposure to sunlight (full sun, partial sun, or shade) and any signs of visible surface wetness were recorded. Entrances fully covered in impervious surface were not sampled.

We also collected human and animal fecal material from study compounds to validate candidate MST assays. Fresh animal feces that could be attributed to specific individuals were collected as available during any sampling visit using a disinfected metal scoop to transfer individual stools into a sterile sample bag. To obtain human-source fecal material, we sampled fecal sludge from traditional shared latrines during separate visits in February 2016. Each latrine sludge sample was collected in a sterile 50 mL centrifuge tube that had been attached to a metal handle of sufficient length to lower through a latrine drophole to reach the sludge surface. This apparatus was used to scrape sludge into the tube from at least three locations on the sludge surface to collect a composite sample.

### 2.4 Validation of microbial source tracking assays

#### 2.4.1 Identification of candidate MST assays

We considered eight open-source qPCR assays targeting general, human, and avian fecal microbes to assemble a panel of candidate MST markers (Table 1). Preference was given to assays previously validated in multi-laboratory comparison studies, as well as to ensuring a variety of organisms and gene targets were represented among the candidates (Johnston et al., 2013; Layton et al., 2013). We prioritized human source-associated assays in light of the associated intervention trial intended to reduce human fecal contamination, and considered avian-associated assays owing to the frequent observation of chickens and ducks in study compounds. While cats, dogs, goats, and pigs were also observed with varying frequency, we were unable to collect sufficient fecal samples from these sources to adequately validate any other animal-associated assays. We considered assays for non-host specific fecal microbes as a basis for relating molecular detection to culture-based detection of general fecal indicator bacteria.

**Table 1:** Candidate qPCR assays for microbial source tracking.

ID^a^	assay	host	organism/gene	class	detection
BacUni	BacUni-UCD (Kildare et al., 2007)	general	*Bacteroidales* 16S	bacterium	probe
EC23S	EC23S857 (Chern et al., 2011)	general	*E. coli* 23S	bacterium	probe
BacHum	BacHum-UCD (Kildare et al., 2007)	human	*Bacteroidales* 16S	bacterium	probe
HAdV	HAdV (Jothikumar et al., 2005)	human	Adenovirus hexon gene	virus	probe
HF183	HF183/BacR287 (Green et al., 2014)	human	*B. dorei* 16S	bacterium	probe
Mnif	Mnif (Johnston et al., 2010)	human	*M. smithii nifH*	archaeon	probe
GFD	GFD (Green et al., 2012)	avian	*Helicobacter* spp.	bacterium	SYBR
LA35	LA35 (Weidhaas et al., 2010)	avian	*Brevibacterium* sp. 16S	bacterium	SYBR

a Abbreviated assay identifier used throughout the text.

#### 2.4.2 Validation qPCR

DNA was extracted from animal feces and latrine sludge in Maputo using the FastPrep SPIN Kit for Soils (MP Biomedicals, Santa Ana, CA, USA) and stabilized with DNAstable Plus (Biomatrica, San Diego, CA, USA) for ambient temperature transport to the US for further analysis (Eichmiller et al., 2016; Pontiroli et al., 2011). We validated the candidate assays against each fecal sample using singleplex qPCR. All validation reactions consisted of 12.5 μL TaqMan Environmental Master Mix 2.0 (Applied Biosystems, Foster City, CA, USA), 2.5 μL 10x primers and probe mix, and 10 μL of diluted DNA template, for a total reaction volume of 25 μL (Odagiri et al., 2015). All probes were labeled with 6-FAM reporter dye and BHQ-1 quenchers except for the BacUni and HF183 probes, which were labeled with BHQplus quenchers (LGC Biosearch Technologies, Middlesex, UK). Reactions were performed on a CFX96 Touch thermocycler (Bio-Rad, Hercules, CA) with an initial 10-min incubation at 95 °C, followed by cycles of denaturation and annealing for the durations, temperatures, and cycle numbers described in the original published protocol for each assay (Table S1). Both ten-fold and hundred-fold sample dilutions were used as DNA template to account for potential PCR inhibition (Odagiri et al., 2015). We ran each sample dilution in duplicate, including duplicate ten-fold standard dilution series from 10^7^ – 10^1^ copies (gc) of artificial plasmid standard (Table S2) and four non-template control (NTC) reactions on each instrument run. Raw qPCR output was processed using CFX Manager software (Bio-Rad) to calculate quantification cycle (Cq) values using the baseline subtraction method with a 100 RFU florescence threshold (Cao et al., 2012; Layton et al., 2013).

#### 2.4.3 Assay performance evaluation

We evaluated candidate assays primarily on the basis of binary diagnostic performance. The microbial target of a given assay was considered detected in reactions producing a Cq value lower than an assay-specific cutoff point. We considered a fecal sample positive for a given microbial target if the target was detected in any reactions containing DNA template from the sample. Latrine sludge samples were used as human fecal sources, duck and chicken samples represented avian fecal sources, and dog and pig fecal samples were non-target sources for all host-associated assays. General fecal assays were considered associated with all fecal samples for the purposes of performance evaluation.

To reduce the potential for false positives from amplification artifacts, we used receiver operator characteristic (ROC) analysis to obtain assay-specific cycle cut-off points for determining reaction detection status (Nutz et al., 2011). ROC curves were generated for cycle cutoffs in one-Cq increments from 10 Cq to the maximum number of cycles described by the assay developers. Reactions with Cq values below the cutoff point were classified as positive and above the point as negative. Diagnostic sensitivity and specificity were calculated from all reactions (including extraction blanks) at each cutoff point (see Supplementary Material). The highest whole Cq value that maximized the Youden index, computed as J=sensitivity+specificity−1, was selected as the optimal cutoff point for each assay (Fluss et al., 2005; Nutz et al., 2011).

### 2.5 Microbial analysis of environmental samples

#### 2.5.1 Filtering and culture-based analysis

Environmental samples were processed by membrane filtration prior to further microbial analysis. We filtered water samples and eluate from surface swabs and soil samples as described in the Supplementary Material. Culture-based enumeration of *E. coli* was performed following a modification of USEPA Method 1603 on 0.45 μm cellulose ester membranes (MilliporeSigma, Burlington, MA, USA) (USEPA, 2009). We filtered 100 mL and 10 mL volumes of water samples and 1 ml and 0.1 mL of surface swab and soil eluate. Up to 300 mL water, 12 ml swab eluate, and 30 mL soil eluate were filtered through 0.4 μm polycarbonate membranes (MilliporeSigma) and immediately stored at −80 °C for molecular analysis. Excess water and eluate were retained at 4 °C until the plates were read following 22–26 h incubation, over which time minimal *E. coli* die-off would be expected in the stored samples (Harmel et al., 2016; Pope et al., 2003). If the lowest volume plate for a given sample was too numerous to count (TNTC), we filtered an additional 1 mL water sample and 0.01 mL for swab or soil eluate.

Soil moisture content was determined by drying approximately 5 g wet soil by microwave oven in 5-min increments until the measured weight stabilized. The soil moisture fraction is given by the difference between the initial and final weights divided by the initial sample weight (Capone et al., 2019; Pickering et al., 2012).

#### 2.5.2 DNA isolation from filtered samples

We extracted DNA from soil and swab sample filters with the DNeasy PowerSoil kit (Qiagen, Hilden, Germany), which yielded DNA free of PCR inhibitors in previous comparison studies (Eichmiller et al., 2016; Mahmoudi et al., 2011). Anticipating lower DNA abundance in water samples, we extracted DNA from water sample filters with the DNA-EZ ST01 kit (GeneRite, North Brunswick, NJ, USA), which previously demonstrated higher DNA yields from marine water samples than the PowerSoil kit (Cox and Goodwin, 2013). To further address potential inhibition and provide a specimen processing control (SPC), 3 μg salmon testes DNA (MilliporeSigma) was added to all extraction bead tubes prior to loading sample filters (Haugland et al., 2005, 2012). In each extraction batch, two tubes were filled with blank filters to serve as negative and positive extraction controls (NEC and PC respectively). We spiked the PC tubes with $2\times 10^{8}$ copies of each artificial plasmid standard (Table S2). Samples were lysed with a Mini-Beadbeater (BioSpec, Bartlesville, OK, USA) at maximum speed for 120 s, after which we followed the PowerSoil manufacturer protocol or the Source Identification Protocol Project DNA-EZ ST01 protocol as appropriate (Boehm et al., 2013; Griffith et al., 2013). Purified DNA was eluted with 100 μL elution buffer, aliquoted in 25 μL volumes, and immediately stored at −80 °C, retaining one aliquot for further evaluation. The remaining aliquot was stored at 4 °C for up to 72 h before measuring DNA concentration with a NanoDrop Lite spectrophotometer (Thermo Scientific, Waltham, MA, USA) and testing for PCR inhibitors.

#### 2.5.3 Molecular detection of microbial targets

We assessed microbial targets in environmental samples using four qPCR assays selected from the candidate set—EC23S, HF183, Mnif, and GFD—and assessed PCR inhibition with a fifth qPCR assay, Sketa22, targeting the salmon testes DNA SPC. Each reaction consisted of 12.5 μL TaqMan Environmental Master Mix 2.0, 2.5 μL 10x primers and probe mix, 5 μL nuclease free water (NFW), and 5 μL of DNA template, for a total reaction volume of 25 μL. Cycling conditions were identical to the validation analysis. Template DNA was used undiluted unless a specific sample was determined to be inhibited, in which case DNA was diluted five-fold (Haugland et al., 2012). We considered a sample to be inhibited if the Cq value for the Sketa22 assay was >3 Cq above the mean Sketa22 Cq of its associated extraction controls (both NEC and PC) (Gentry-Shields et al., 2012; Haugland et al., 2012). Reactions were performed in duplicate for 10% of samples selected randomly within the set of samples of each type. Each 96-well reaction plate typically contained samples from three extraction batches, resulting in three NECs and three PCs per plate, as well as three NTC reactions. We prepared five-point, ten-fold dilution series from each of the three PCs on a given plate, corresponding to triplicate reactions of 10^5^–10^1^ copies of each artificial plasmid standard before DNA extraction. All samples analyzed on the same plate were extracted by the same method.

#### 2.5.4 Calibration curve construction

We estimated microbial target abundance in environmental samples from observed Cq values using calibration curves fit to known concentrations of standard reference material. The serial dilutions of extracted PCs, analyzed alongside environmental samples as described above, correspond to known concentrations before DNA isolation procedures to account for extraction loss. Calibration curves were fit for each target using multilevel Bayesian regression to account for possible variation between reaction sets (Sivaganesan et al., 2010, 2008). We treated reaction Cq as the response, log_10_ copy number as a predictor, and allowed slopes and intercepts to vary by instrument run and by extraction batch. We fit models with the **brms** package in **R** version 3.5.1, using the default, improper flat priors on population-level coefficients and four chains with 2000 warmup iterations and 2000 sampling iterations (Bürkner, 2018, 2017; R Core Team, 2018).

#### 2.5.5 Microbial target quantification

We quantified culturable *E. coli* (cEC) as colony forming units (cfu) on individual plates and molecular targets as gene copies (gc) in individual reactions. The sampling effort represented by each plate/reaction was used to compute target concentrations in environmental samples, normalized to 100 mL of water, 100 cm^2^ of food preparation surface, or gram of dry soil. We considered each mL of soil or swab sample eluate filtered to represent 0.01 g wet soil and 8.33 cm^2^ surface area, respectively, and each reaction—containing 5 μL of the total 100 μL purified DNA eluted from each filter—to represent 1/20th of the filtered volume. Moisture content was used to normalize soil sampling efforts in terms of dry weight. We imputed missing moisture contents from observations of sun exposure, soil surface wetness, and precipitation, temperature, and wind conditions using multivariate imputation by chained equations (MICE) in the **R** package **mice** (Buuren and Groothuis-Oudshoorn, 2011). We calculated cEC abundance and sampling effort by summing the cfu counts and volumes filtered for all countable plates from a given sample (Levy et al., 2012).

We estimated molecular target concentration distributions from the calibration curve posterior draws to account for uncertainty in the concentration estimates (Gelman and Hill, 2007; McElreath, 2015). At each sampling iteration, we estimated target log_10_ gc in each reaction using the extraction batch- and instrument run-specific slope and intercept parameter values. We normalized the reaction log_10_ gc estimates by sampling effort and combined the transformed posterior draws from all replicate reactions to construct the posterior distribution of target concentration in each environmental sample. Target concentrations were characterized as the mean, standard deviation (SD), and 2.5 and 97.5 percentiles (95% credible interval) of the log_10_ concentration posterior distributions.

#### 2.5.6 Determining limits of detection

For each sample and microbial target, we calculated process limits of detection that accounted for the amount of sample processed and potential target loss throughout the analytical procedure, expressed in terms of target concentration in each sample. For cEC, we assumed a lower limit of detection (LLoD) of one cfu per plate and an upper limit of quantification (ULoQ) of 400 cfu per plate, as suggested by Levy et al. (2012) and supported by raw counts from our samples. We obtained the process limits in terms of cEC concentration using the largest volume filtered for samples with no growth on any plate and the smallest volume filtered for samples with all TNTC plates. This corresponds to process LLoDs of 1 cfu/100 mL water, 12 cfu/100 cm^2^ surface, and 100 cfu/g wet soil (109 cfu/g dry soil for a moisture content of 8.4%, the median of all soil samples collected), and process ULoQs of 4.6 log_10_ cfu/100 mL water, 5.7 log_10_ cfu/100 cm^2^ surface, and 6.6 log_10_ cfu/g wet soil (6.64 log_10_ cfu/g dry soil at median soil moisture content). We defined process LLoDs for molecular targets as the log_10_ concentrations corresponding to the ROC-derived cutoff Cq value for each assay. Target concentrations at the cutoff Cq values were estimated for each sample using sample-specific sampling efforts and calibration curve posterior draws.

#### 2.5.7 Treatment of observations outside detection and quantification limits

When analyzing binary detection outcomes, we treated observations below the LLoD as negative and observations above the LLoD, including > ULoQ, as positive. For continuous concentration outcomes, we treated observations below the LLoD and above the ULoQ as left and right censored, respectively. We obtained maximum likelihood estimates (MLE) of the log_10_ concentration mean and SD assuming a censored normal distribution with the *fitdistcens* function in the **R** package **fitdistrplus** (Delignette-Muller and Dutang, 2015). We also imputed concentrations for censored observations as the expected value of a normal distribution truncated at the sample-specific LLoD or ULoQ using the *etruncnorm* function from the **R** package **truncnorm** and the MLE mean and SD (Mersmann et al., 2018; Messier et al., 2012).

### 2.6 Assessing risk factors of domestic fecal contamination

#### 2.6.1 Risk factor data sources

Compound- and household-level socioeconomic, demographic, sanitary, and health characteristics were ascertained by trained local enumerators using surveys and direct observation as described previously (Knee et al., 2018). Surveys were conducted in Portuguese or Changana, a local language, according respondent preference. We identified characteristics that presented potential fecal contamination hazards, household and compound amenities that could affect domestic fecal microbe transmission, and demographic characteristics that may be indicative of the resources available for, and challenges to, managing domestic fecal wastes (Table S5). GPS-enabled tablets displaying orthorectified, geolocated satellite imagery were used to delineate compound boundaries, from which we calculated compound area and population density. Daily meteorology records were obtained for the weather station at Maputo International Airport, located adjacent to our study area, from the Global Surface Summary of Day dataset available through the National Oceanic and Atmospheric Administration's National Centers for Environmental Information (https://www7.ncdc.noaa.gov/CDO/cdoselect.cmd). Physical characteristics of each sample were observed during collection or determined during initial laboratory processing, in the case of soil moisture. Additional information about the variables assessed for the risk factor analysis is available in the Supplementary Material.

#### 2.6.2 Statistical analysis

We used univariable analyses to test associations between each putative risk factor and occurrence of microbial targets. Separate analyses were performed for each sample type to allow for different patterns of fecal contamination. The number of independent comparisons entailed by this approach increases the probability of Type I error, likely resulting in the observation of some number of spurious associations (Gelman et al., 2012), but the broader pattern of associations can be suggestive of conditions and processes related to domestic fecal contamination that may warrant further investigation. Normalized log_10_ target concentration was used as the response variable unless the target was detected in <75% of samples of a given type, in which case the binary detection status served as the response. Continuous variables other than cumulative precipitation were mean-centered and scaled, either by SD (i.e., standardized) or by a meaningful value for the particular variable (e.g., wealth index scaled such that each unit-increase represented a 10-point increase on the original 0–100 index scale). Due to infrequent precipitation, we represented precipitation variables as cumulative sums over both the seven and 30 days preceding the sampling event to obtain positive values and investigate different temporal scales (Holcomb et al., 2018). Variable definitions, including categorical variable reference categories, are provided in Table S5. We estimated associations for concentration responses with censored Bayesian regression to account for observations outside the limits of detection and quantification, which provides a measure of effect in terms of the change in target log_10_ concentration for a unit increase in the risk factor (Stan Development Team, 2019a). Population-level parameters were assigned weakly regularizing normal priors with SD = 10 for the intercept and SD = 2 for predictors (McElreath, 2015). Bayesian logistic regression was used for binary responses with the odds ratio (OR) serving as the measure of effect. Weakly regularizing Student's *t* priors with 5 degrees of freedom were assigned to population-level parameters, using scale = 10 for the intercept and scale = 2.5 for predictors (Gelman et al., 2008; Stan Development Team, 2019b). The strength of estimated associations was primarily characterized by whether the 95% CIs excluded the null value for their respective measures of effect. When modeling responses in stored water, food surfaces, and household soil, which were collected from multiple households per compound, the intercept was allowed to vary by compound to account for clustering of observations. Models were fit in **brms** using four chains with 1500 warmup and 1000 sampling iterations each.

## 3 Results

### 3.1 Candidate assay diagnostic performance

Individual local fecal samples were collected from 10 chickens, 13 ducks, one dog, and two pigs, as well as a composite manure sample from 6 piglets. Surface sludge was obtained from 14 unimproved pit latrines, representing composite human-source fecal material. We analyzed each sample with eight qPCR assays to assess diagnostic performance, implementing ROC analysis to determine the optimal cutoff Cq value for each assay. Table 2 presents the ROC-derived optimal cutoff cycle and the corresponding sensitivity, specificity, and accuracy for each assay. Both general assays performed well, though EC23S was positive for 100% of samples while BacUni was negative for a single chicken sample. Host-associated assays were all reasonably specific, ranging from 71% (HF183) to 100% (GFD). All human assays cross-reacted with avian feces, though not to the extent seen in certain previous studies (Harris et al., 2016; Odagiri et al., 2015). HAdV was the most human-specific, cross-reacting with only two duck fecal samples; BacHum and Mnif were both positive for certain chicken and duck samples, while HF183 cross-reacted with chickens, ducks, and a pig sample. All assays were negative for the dog sample with the exception of LA35, which was also positive for a single latrine sample.

**Table 2:** Optimal cutoff cycle and diagnostic performance of candidate MST assays.

assay	host	cutoff cycle	test samples	target samples	non-target samples	sensitivity	specificity	accuracy
BacUni	general	38	41	41	0	0.95	–	–
EC23S	general	39	41	41	0	1.00	–	–
BacHum	human	40	41	14	27	0.50	0.81	0.71
HF183	human	39	41	14	27	0.64	0.67	0.66
Mnif	human	41	41	14	27	0.71	0.70	0.71
HAdV	human	44	41	14	27	0.79	0.93	0.88
LA35	avian	45	41	23	18	0.43	0.89	0.63
GFD	avian	40	41	23	18	0.78	1.00	0.88

Sensitivity was lower than specificity for all host-associated assays. HAdV was the most sensitive human marker (79%), followed by Mnif (71%) and HF183 (64%). BacHum, while the second-most specific human-associated target, was only detected in half the human samples. GFD was substantially more sensitive (78%) than LA35, which was positive in fewer than half the avian samples.

We anticipated further reductions in assay sensitivity and improvements in specificity when applied to environmental samples, expecting dilution effects to result in lower ambient concentrations of fecal microbes than in whole feces or sludge. We therefore weighted sensitivity more highly when selecting assays for use in MST analysis of environmental samples. We selected EC23S as general fecal target, both for improved sensitivity relative to BacUni and to provide a molecular comparison to the cultured *E. coli* data. GFD was both more sensitive and specific than LA35 and was chosen for the avian target. Despite relatively strong specificity, we excluded BacHum due to low sensitivity. Although HAdV demonstrated relatively high sensitivity in our samples, its sensitivity in a much larger study was very poor (Harwood et al., 2013), presenting substantial uncertainty about its continued performance across the study area and throughout the study period, particularly in ambient samples. Accordingly, we selected Mnif and HF183 as human targets for further MST analysis.

### 3.2 Occurrence of fecal indicator organisms in the domestic environment

#### 3.2.1 Environmental sample characteristics

We collected 366 samples from 94 households in 58 compounds, home to 135 children previously enrolled in the MapSan trial. Samples were collected on 27 (noncontiguous) days. Source water was available for collection in only 44 compounds, with some compounds lacking water points and the municipal supply intermittently unavailable during the sampling visits. Soil was collected from 56 compound latrine entrances and 85 household entrances; soil could not be collected from eight households in five compounds due to impervious surfaces surrounding the household entrances. Moisture content was missing for three latrine soil and four household soil samples and was imputed using MICE. We collected stored water and food preparation surface swabs from 91 and 90 households, respectively, with two sets of swabs collected from 89% (80/90) of food preparation surfaces.

Soils were generally shaded to some extent and often had wet surfaces (Table 3), though latrine soils were somewhat more commonly exposed to full sun (27%) and wet (67%) than household soils (19% and 57%, respectively). Mean moisture content was nevertheless similar between soil samples from both locations. Plastic bowls comprised the large majority of food preparation surfaces sampled; nearly every water storage container was likewise constructed of plastic (92%). Storage containers typically had wide mouths (71%) with lids (70%), from which water was extracted by dipping a cup or pitcher inside the container. Conversely, water was typically poured out of narrow-mouthed containers, which were generally observed uncovered.

**Table 3:** Number (%) of samples observed with a given characteristic.

type	characteristic	value	n	observations (%)
stored water	container material	plastic	91	84 (92)
		metal		4 (4)
		other		3 (3)
	container opening	covered	89	62 (70)
		uncovered		27 (30)
	container mouth	wide	89	63 (71)
		narrow		26 (29)
	extraction method	dip	89	62 (70)
		pour		27 (30)
food surface	type	bowl	90	83 (92)
		table		7 (8)
	material	plastic	90	79 (88)
		metal		8 (9)
		wood		3 (3)
latrine soil	sun exposure	full	49	13 (27)
		partial		34 (69)
		shaded		2 (4)
	surface wetness	dry	49	16 (33)
		wet		33 (67)
	moisture content	percent^a^	56	9.4 (8.4)
household soil	sun exposure	full	85	16 (19)
		partial		52 (61)
		shaded		17 (20)
	surface wetness	dry	83	36 (43)
		wet		47 (57)
	moisture content	percent^a^	85	8.4 (8.1)

a As mean (IQR) of sample values.

#### 3.2.2 Process limits of detection

The minimum concentration at which each molecular target could be reliably detected was estimated for each sample from the corresponding extracted PC calibration curve (Table S3). The volume filtered for qPCR detection was consistent between samples of the same matrix (e.g., water), although normalizing by dry weight introduced variability into the amount of each soil sample processed. Analytical sensitivity varied by assay, extraction batch, and instrument run. For a given target, average sample LLoDs were similar between samples of the same matrix (Table 4). GFD consistently demonstrated the lowest detection limits while average LLoDs for EC23S, HF183, and Mnif were generally similar for a given sample type. This likely reflects higher analytical sensitivity on the part of GFD, reflected in the lower intercept values for the GFD calibration curves (Table S3), which used non-specific SYBR chemistry for real-time detection. The LLoD estimates for the probe-based assays indicate relatively high concentrations were necessary for reliable detection, generally requiring more than 1000 target copies per 100 mL water or 100 cm^2^ surface and >10,000 per gram of soil.

**Table 4:** Mean (SD) process lower limits of detection for molecular targets.

sample type	n^a^	EC23S	HF183	Mnif^a^	GFD
source water [log_10_ gc/100 mL]	44	3.38 (0.04)	2.77 (0.35)	3.14 (0.10)	2.41 (0.20)
stored water [log_10_ gc/100 mL]	91	3.36 (0.10)	3.04 (0.35)	3.11 (0.07)	2.30 (0.04)
food surface [log_10_ gc/100 cm^2^]	89	3.91 (0.42)	3.50 (0.47)	3.52 (0.50)	2.83 (0.49)
latrine soil [log_10_ gc/dry g]	56	4.59 (0.11)	4.24 (0.19)	4.12 (0.16)	3.36 (0.17)
household soil [log_10_ gc/dry g]	84	4.50 (0.14)	4.45 (0.19)	4.34 (0.13)	3.31 (0.39)

a Sample number; due to improper amplification, one sample each of stored water, food surface, and household soil were excluded from the analysis of Mnif.

#### 3.2.3 Microbial target detection frequency

We detected at least one microbial target in 96% of samples (353/366). Highly credible *E. coli*, detected by both culture and qPCR, were present in the majority of samples (78%). EC23S and cEC were detected with similar frequency except in source water (Table 5), in which *E. coli* was detected twice as frequently by qPCR (66%) than culture (34%). Human targets were frequently detected in soils (59%) and occasionally in stored water (17%) but seldom in source water or on food surfaces. Mnif was more common than HF183 in both latrine and household soil, though HF183 was the only human marker detected with any frequency in samples other than soil. We observed the largest human fecal impact on latrine soil, with 68% positive for at least one human target and 27% positive for both, an indicator of highly credible human-source contamination. The avian target GFD was rarely detected in any sample type but was most common in soils (4%). The lowest detection frequencies for all targets were observed in source water, the sample type with the shortest residence time on the compound premises.

**Table 5:** Fraction (%) of samples positive for each target by sample type.

target	source water	stored water	food surface	latrine soil	household soil
cEC	15/44 (34)	81/91 (89)	81/90 (90)	54/54 (100)	85/85 (100)
EC23S	29/44 (66)	79/91 (87)	75/89 (84)	53/56 (95)	84/84 (100)
any *E. coli*	34/44 (77)	90/91 (99)	89/90 (99)	55/55 (100)	85/85 (100)
both *E. coli*	10/44 (23)	70/91 (77)	67/89 (75)	52/55 (95)	84/84 (100)
HF183	1/44 (2)	15/91 (16)	1/89 (1)	21/56 (38)	21/84 (25)
Mnif	0/44 (0)	1/90 (1)	1/88 (1)	32/56 (57)	29/83 (35)
any human	1/44 (2)	15/90 (17)	2/88 (2)	38/56 (68)	45/84 (54)
both human	0/44 (0)	1/91 (1)	0/89 (0)	15/56 (27)	5/83 (6)
GFD	0/44 (0)	1/91 (1)	0/89 (0)	2/56 (4)	3/84 (4)
any target	34/44 (77)	90/91 (99)	89/90 (99)	55/55 (100)	85/85 (100)

All laboratory blanks were culture-negative for cEC (n = 68) and all NTC reactions were negative for HF183 (n = 18), Mnif (n = 15), and GFD (n = 18). Likewise, all reactions containing NECs were negative for HF183 (n = 23), Mnif (n = 23) and GFD (n = 23). However, 6% of NTC reactions (1/17) and 17% of NEC reactions (4/23) were positive for EC23S, with a mean Cq of 38.1 and a minimum Cq of 37.1. Such values only slightly exceed the EC23S optimal cutoff cycle of 39 and are consistent with the low levels of DNA contamination that others have observed for this assay and have attributed to residual *E. coli* DNA in the Environmental Master Mix arising from the production of *Taq* polymerase (Shrestha and Dorevitch, 2019). Sketa22 analysis indicated inhibition in a single latrine soil sample, which was diluted five-fold for all further qPCR analyses (Haugland et al., 2012). Duplicate reactions were assessed for molecular targets in 36 randomly selected samples (10% of each type). The same EC23S detection status was observed in both replicates for 97% of these samples, with agreement between replicates on HF183 presence for 86% of samples, agreement on Mnif for 91% of samples, and agreement on GFD for 94% of samples. The reduced agreement between replicates for HF183, and to a lesser extent for Mnif, may partially reflect the greater variability in human target detection relative to EC23S, which was nearly always present, and GFD, which was usually absent.

#### 3.2.4 Microbial target concentrations

Assuming log_10_ target concentrations followed a normal distribution with left- and right-censored observations, we obtained the MLE mean and SD concentration of each target detected in >10% of a given sample type (Table 6). Because we normalized according to the matrix sampled, concentrations may be directly compared between samples of the same matrix (e.g. source water and stored water) but not between matrices. Furthermore, while each cfu is assumed to correspond to a single organism present in the sample, organisms may carry different numbers of each gene target, limiting comparability between targets. We assessed cEC and EC23S concentrations in all sample types, HF183 concentration in stored water and soils, and Mnif concentration in soils only; GFD was detected too infrequently in any sample to characterize concentration.

**Table 6:** Maximum likelihood estimate (SE) of target concentration mean and SD under a normal distribution with censored observations.

type^a^	cEC			EC23S			HF183			Mnif		
	n	mean	SD	n	mean	SD	n	mean	SD	n	mean	SD
source water [log_10_C/100 mL]	44	−0.54 (0.33)	1.38 (0.29)	44	3.57 (0.14)	0.84 (0.12)						
stored water [log_10_C/100 mL]	91	1.72 (0.16)	1.46 (0.12)	91	4.26 (0.09)	0.83 (0.07)	91	1.39 (0.48)	1.66 (0.36)			
food surface [log_10_C/100 cm^2^]	90	3.17 (0.21)	1.95 (0.18)	89	4.73 (0.10)	0.90 (0.08)						
latrine soil [log_10_C/dry g]	54	3.95 (0.13)	0.97 (0.10)	56	6.48 (0.16)	1.17 (0.12)	56	3.79 (0.27)	1.39 (0.25)	56	4.31 (0.17)	1.11 (0.15)
household soil [log_10_C/dry g]	85	4.14 (0.10)	0.90 (0.07)	84	6.72 (0.10)	0.88 (0.07)	84	3.31 (0.36)	1.65 (0.30)	83	4.11 (0.10)	0.57 (0.09)

a Units for each sample type expressed in brackets, where C represents cfu or gc as appropriate.

*E. coli* gene targets were more abundant than human targets. Mean EC23S and HF183 concentrations were respectively 6.5 and 3.8 log_10_ gc/dry g of latrine soil and 4.3 and 1.4 log_10_ gc/100 ml of stored water. The censoring assumption implies that non-detected targets were not absent but rather present in concentrations too low for reliable detection, reflected in mean concentration estimates for human targets below their estimated LLoDs (Table 4). By contrast, EC23S, which was detected in nearly every sample, had mean concentrations well above its mean LLoD for each sample type. Among the 34% of source water samples positive for cEC, the mean concentration was 8.5 cfu/100 mL.

### 3.3 Domestic risk factors of fecal contamination

#### 3.3.1 Characteristics of study households, compounds, and sampling dates

All compounds had a latrine on premises and most covered the latrine drophole (86%), but otherwise latrine quality was poor: only 36% had a slab and 26% a permanent superstructure (Table 7). Most compounds (82%) had water sources on premises, though source water was only available in 70% at the time of sampling. Electricity was nearly universally available, and most households (97%) had impervious floors. Potential fecal hazards were present in many compounds, with standing wastewater observed in 58% of compounds, 56% owning domestic animals, and 49% reporting previous flooding. Additionally, disposal of feces outside the latrine was reported for at least one child in 86% of compounds.

**Table 7:** Summary of household, compound, and sampling date characteristics; binary outcomes as positive observations (%) and continuous outcomes as mean (IQR).

		level	n	observations
*hazards*				
feces or soiled diapers observed		compound	57	21 (37)
standing wastewater observed		compound	57	33 (58)
prone to flooding		compound	57	28 (49)
animals present	any	compound	57	32 (56)
poultry			57	8 (14)
cat			57	27 (47)
dog			57	6 (11)
other			57	2 (4)
reported child diarrhea		household	90	8 (9)
in any household		compound	57	6 (11)
unsafe child feces disposal		household	90	72 (80)
in any household		compound	57	49 (86)
*amenities*				
latrine on premises		compound	57	57 (100)
cabins (count)			57	1.0 (0.0)
drophole cover			57	49 (86)
slab or pedestal			57	22 (39)
superstructure			57	15 (26)
ventpipe			57	1 (2)
water on premises		compound	57	47 (82)
water points (count)		compound	57	1.4 (1.0)
household-reported access		household	91	79 (87)
available during sampling		compound	57	40 (70)
electricity on premises		compound	57	55 (96)
covered floor		household	91	88 (97)
*demographics*				
completed primary education	head of household	household	91	28 (31)
child caregiver		household	90	49 (54)
child caregiver in any household		compound	57	41 (72)
wealth index (0–100)		household	91	45.8 (12.2)
household members (count)		household	91	6.2 (3.5)
children enrolled			90	1.3 (1.0)
rooms in house (count)		household	91	2.8 (1.0)
persons per room (ratio)		household	91	2.3 (1.3)
crowding (>3)			91	12 (13)
compound population (count)		compound	57	17.3 (7.0)
children enrolled			57	2.2 (2.0)
households enrolled			57	1.7 (1.0)
compound area (m^2^)		compound	52	279.5 (156.3)
population density (persons/100 m^2^)		compound	52	7.2 (5.1)
persons per latrine (ratio)		compound	57	17.2 (7.0)
persons per water point (ratio)		compound	47	12.4 (7.8)
*meteorology*				
temperature previous day (°C)	mean	date	27	20.4 (1.7)
minimum			27	14.1 (3.2)
maximum			27	28.2 (4.4)
windspeed previous day (knots)		date	27	7.7 (3.2)
cumulative precipitation (mm)	previous day	date	26	2.8 (0.0)
previous week			27	9.3 (3.0)
previous month			27	41.6 (57.1)
days with any rain (count)	previous seven days	date	27	0.6 (1.5)
previous 30 days			27	2.5 (4.0)

The average compound had 17.3 members and 2.2 children enrolled in the study from 1.7 households, each of which had an average of 6.2 members and 1.3 enrolled children. About half of child caregivers (primarily mothers) reported completing primary school, though fewer household heads had done so (31%). The wealth of most households fell within the middle range of the 100-point asset-based index, with the typical household slightly below the index midpoint with a value of 46. Few households (12%) were crowded with more than three people per room. Compounds had a mean area of 280 m^2^ and population density of 7.2 people/100 m^2^. The weather during sampling was relatively dry and mild, with daily average temperatures of 20 °C. On the average sampling day it had rained a total of 4.2 cm on 2.5 days in the preceding month.

#### 3.3.2 Risk factor associations with fecal indicator concentrations

**Fig. 1:**
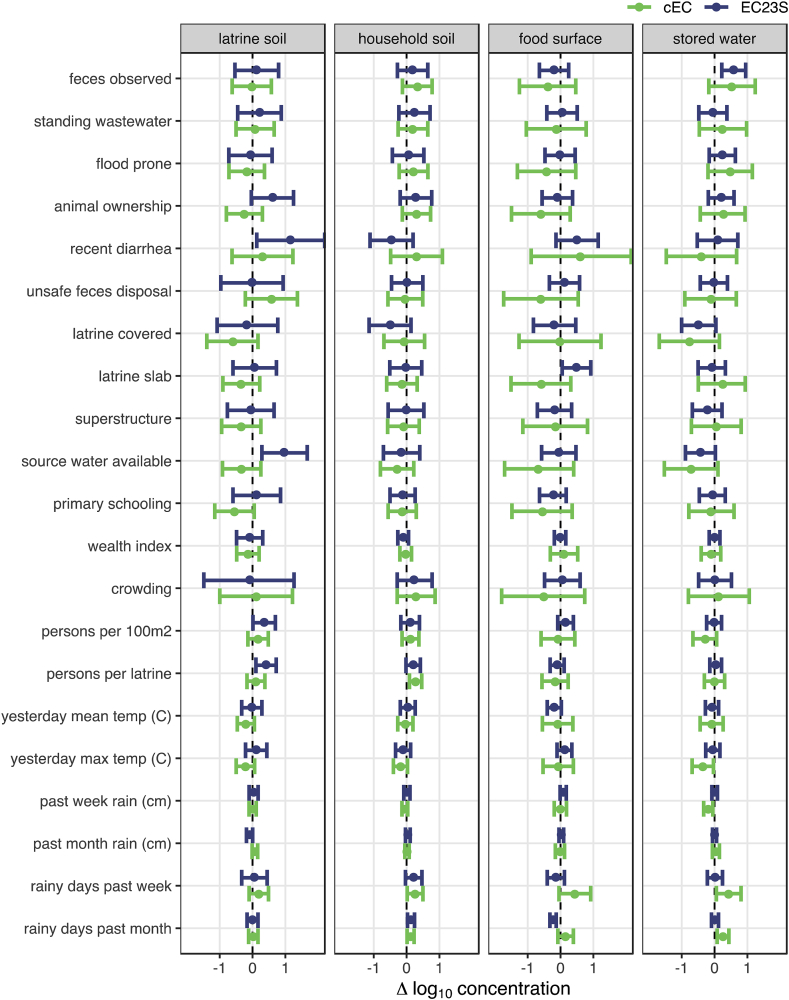
Mean and 95% CI change in log_10_*E. coli* concentrations associated with sanitary, sociodemographic, and meteorological characteristics estimated by multilevel Bayesian censored linear models. 95% CIs that do not include zero suggest fecal contamination risk factors.

We assessed risk factors of general fecal contamination as the expected linear change in normalized log_10_ concentration of cEC and EC23S given the presence of a binary predictor variable or a one unit increase in a scaled continuous variable (see Table S5 for variable definitions). Predictors for which the 95% CI of the effect estimate included zero were considered unlikely to be risk factors of contamination for the sample type tested, though we considered the sign of the point estimate across targets and sample types to evaluate the broader implications of each variable. There were few consistent trends in associations with target concentrations and most characteristics were not clearly associated with either target in most sample types (Fig. 1, Table S6). No compound, household, or meteorological characteristic was significant across all sample types; rather, the direction of the effect estimate often reversed between targets and sample types. cEC concentrations were elevated in all sample types with increased days of rain the previous week and attenuated with increased temperature, but most of these associations were not significant and did not hold for EC23S or for increased cumulative precipitation over the same period of time. Both cEC and EC23S concentrations declined when latrine dropholes were covered, but all the effects were relatively small and not significant. The strongest effects were observed on EC23S concentration in latrine soil, with an expected increase of 1.2 (95% CI: 0.1–2.2) log_10_gc/dry g when at least one child in the compound had diarrhea in the previous week and 1.0 (95% CI: 0.3–1.7) log_10_gc/dry g when source water was available during sampling. EC23S was also elevated by 0.6 (95% CI: 0.2–1.0) log_10_gc/100 mL in stored water when feces were observed.

**Fig. 2:**
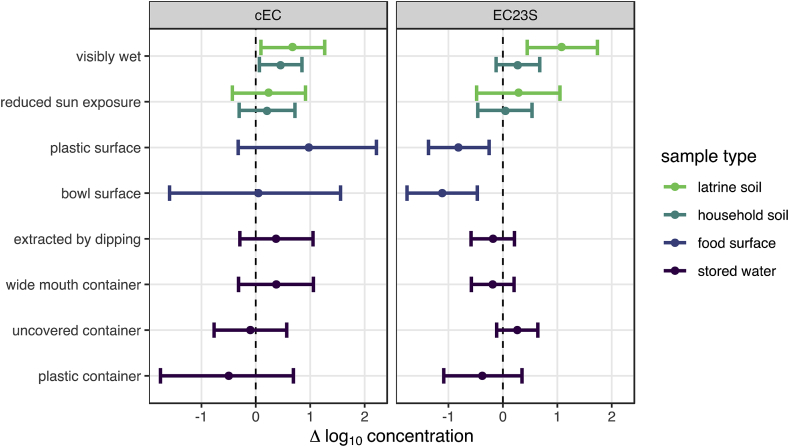
Mean and 95% CI change in log_10_*E. coli* concentrations associated with environmental sample characteristics estimated by multilevel Bayesian censored linear models. 95% CIs that do not include zero suggest fecal contamination risk factors.

Among sample-level characteristics (Fig. 2, Table S7), wet soil surfaces were consistently associated with increased *E. coli* concentrations, significantly so for cEC in both latrine (0.7 [95% CI: 0.1–1.3] log_10_gc/dry g) and household (0.5 [95% CI: 0.1–0.9] log_10_gc/dry g) soils and also for EC23S in latrine soil (1.1 [95% CI: 0.5–1.7] log_10_gc/dry g). Food preparation surface characteristics were also associated with EC23S concentration, which was lower for plastic and bowl-type food preparation surfaces (the most common surfaces) than for metal or wooden and table-like surfaces.

#### 3.3.3 Odds of detecting human fecal contamination

**Fig. 3:**
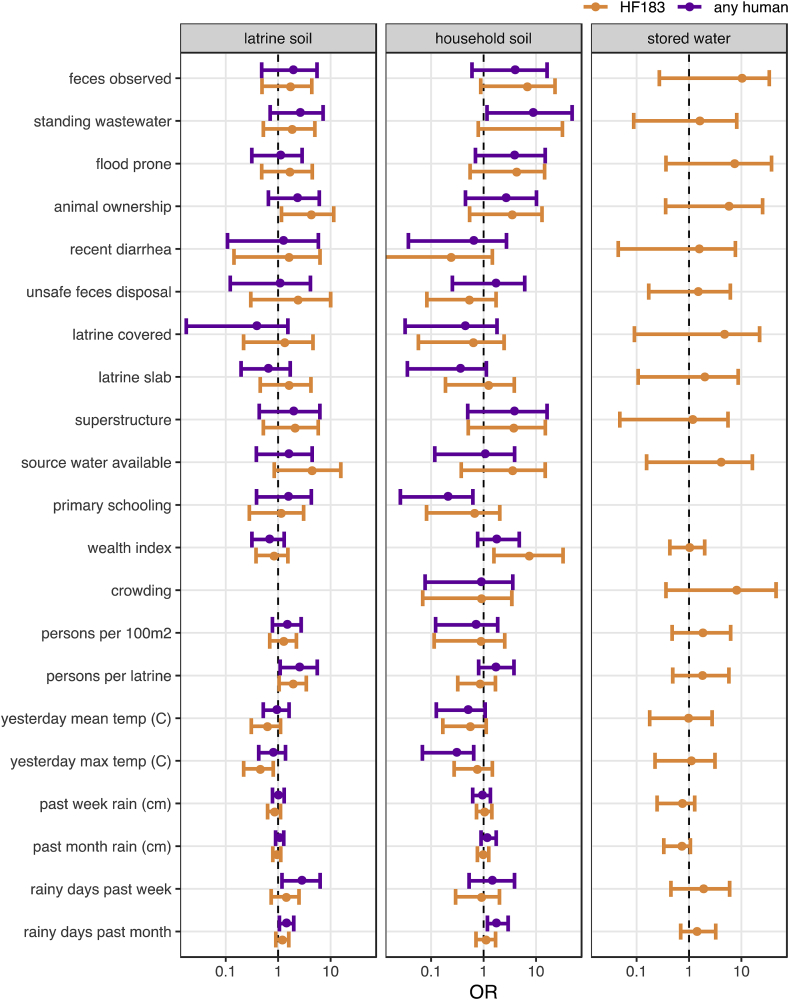
Mean and 95% CI odds ratios of human target detection associated with sanitary, sociodemographic, and meteorological characteristics estimated by multilevel Bayesian logistic models. 95% CIs that do not include unity suggest human fecal contamination risk factors.

Risk factors of human source contamination were identified by the odds ratio for detection of human-associated targets given the presence of a binary predictor variable or a one unit increase in a scaled continuous variable (see Table S5 for variable definitions). We used detection of HF183 and of any human target as response variables in soils and detection of HF183 in stored water, in which Mnif was rarely detected. Human targets were detected too infrequently to assess risk factors for food surface contamination. As with *E. coli* concentrations, we did not find significant associations between most compound, household, and meteorological characteristics and human-source contamination (Fig. 3, Table S6). However, several potential compound hazards were consistently associated with increased ORs for human target detection, including animal ownership, previous flooding, and observation of standing wastewater or feces. The associations were significant, as indicated by 95% CIs on the OR estimate that excluded unity, for HF183 detection in latrine soil when domestic animals were present (OR: 4.3; 95% CI: 1.2–12) and for detecting any human target in household soil in the case of standing wastewater (OR: 8.9; 95% CI: 1.2–49). Completing primary school was associated with reduced odds of detecting any human target (but not HF183 specifically) in household soil (OR: 0.2; 95% CI: 0.03–0.6), while a 10-point increase in the household wealth index was associated with an increase in the odds of human target detection in household soils, significantly so for HF183 (OR: 7.4; 95% CI: 1.6–33). Human contamination was also significantly more common in the soils from latrines as the number of users increased. Increasing temperatures were generally associated with reduced odds of human target detection in soil. Rainy days in the past week and month also usually signaled increased human target detection, though the same association was not present for cumulative precipitation over the same time periods. No variables were significantly associated with detecting HF183 in stored water.

**Fig. 4:**
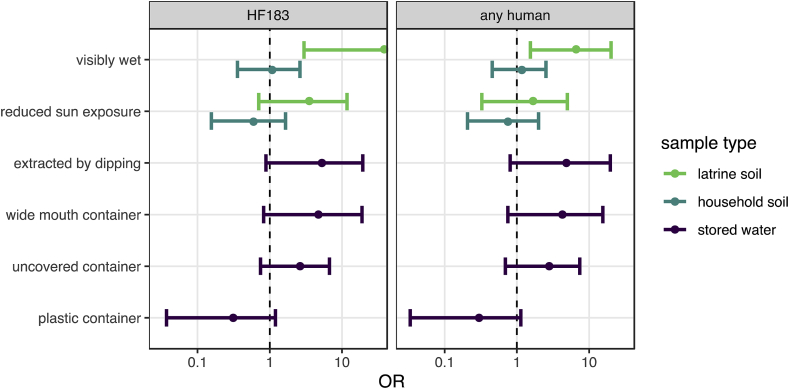
Mean and 95% CI odds ratios of human target detection associated with environmental sample characteristics estimated by multilevel Bayesian logistic models. 95% CIs that do not include unity suggest human fecal contamination risk factors.

The direction of associations between sample characteristics and human target detection were generally similar to those for *E. coli* concentrations (Fig. 4, Table S7). Soil surface wetness effects were less pronounced in household soils for human targets than for *E. coli*, though detection of any human target was significantly more likely in wet latrine soils (OR: 6.6; 95% CI: 1.5–20). Wet latrine soil was also significantly associated with HF183 detection: 18 of 33 wet latrine soils were positive for HF183 and only 1 of 16 dry soils were, which prevented stable estimation of the OR.

## 4 Discussion

We found evidence of widespread fecal contamination, including from human sources, across all environmental compartments sampled. However, compound source water was of moderate quality: two thirds of samples were free from culturable *E. coli*, and the typical concentration in *E. coli*-contaminated samples was less than 10 cfu/100 mL, considered “low risk” under previous WHO drinking water guidelines—though not a distinction with strong support in the literature (Gruber et al., 2014; WHO, 1997). Because source water was treated centrally and generally piped directly into the compound, the elevated prevalence of *E. coli* gene targets relative to culturable *E. coli* may indicate the presence of DNA from organisms inactivated or rendered viable but non-culturable (VBNC) by treatment. We infer from the much higher prevalence of contamination after water had been stored by households that conditions within compounds often led to recontamination of source water after collection (Harris et al., 2019, 2013). Human targets were detected in the 59% of soil samples and 17% of stored water samples, despite relatively low diagnostic sensitivity and high detection limits, suggesting that human-source contamination may have been more pervasive than observed.

The diagnostic performance of all host-associated assays was relatively poor, especially in comparison to their performance in previous multi-site, multi-laboratory studies (Boehm et al., 2013; Layton et al., 2013; Reischer et al., 2013). The exception was human adenovirus assay HAdV, which demonstrated substantially higher sensitivity (with slightly reduced specificity) than previous studies (Harwood et al., 2013). Because all latrine samples were collected over a two-week period, it is possible that we captured a period of elevated adenovirus shedding in the study population and that HAdV sensitivity could be much lower at other times (Lion, 2014). Avian marker GFD also performed relatively well in comparison with the human markers and has been successfully applied in both urban and rural Bangladesh previously (Boehm et al., 2016; Harris et al., 2016). However, we rarely detected GFD in environmental samples despite reported ownership of domestic poultry and frequent observations of poultry and poultry defecation in compound yards while conducting study activities, raising questions about the suitability of GFD in our study area in practice.

In contrast with other domestic MST validation studies that collected fecal samples from individuals (Boehm et al., 2016; Harris et al., 2016; Odagiri et al., 2015), we used latrine sludge to represent human-source feces. The use of fecal sludge in diagnostic performance evaluations introduces additional uncertainty, particularly as inaccurate measures of assay sensitivity and specificity could result if animal feces had been disposed of in the latrine or if the gene targets degraded prior to sampling. However, latrine sludge offers an accessible, non-invasive means of obtaining fecal material from multiple individuals. Composite test samples comprising multiple individual stools are commonly used for MST validation, in part to better capture a range of individual characteristics, such as age, sex, and diet, that may affect MST marker occurrence (Boehm et al., 2016; Harris et al., 2016). Latrines provide natural composite samples, particularly when shared among multiple unrelated households as is common among our study population (Shiras et al., 2018b). We only sampled from the surface of latrines in regular use, on which fresh, minimally mixed feces could typically be observed, limiting the potential for MST marker degradation or contamination from animal sources. Furthermore, GFD was not detected in any latrine sample, suggesting that avian feces were not present at meaningful levels.

While bacterial human MST targets have a fairly stable global distribution in wastewater, their unsatisfactory performance in our study aligns with several other studies that attempted human fecal source tracking in developing contexts to evaluate domestic sanitary conditions (Boehm et al., 2016; Harris et al., 2016; Jenkins et al., 2009; Mayer et al., 2018; Odagiri et al., 2015). Presuming lower abundance of both targets and cross-reacting non-targets in environmental samples compared to feces, we expected diagnostic sensitivity to decline and specificity to increase in practice. That we nevertheless frequently detected human targets while failing to detect avian feces, a known source of cross-reaction (Harris et al., 2016; Odagiri et al., 2015), suggests a heavy burden of human-source fecal contamination in the domestic environment of our study sites. However, we were unable to assess fecal contamination from dogs and cats, the other commonly reported animals among study households, due to a lack of corresponding fecal samples for assay validation. Human MST markers are known to cross-react with dog feces in particular, which we cannot rule out as having produced amplification of human targets in some samples (Layton et al., 2013; Odagiri et al., 2015).

Both non-specific and human-source fecal contamination were largely disconnected from variation in socioeconomic and sanitary conditions within our study population. Although soil FIB concentrations were associated with a sanitary index in a separate subset of the MapSan study population, the small absolute difference in FIB concentrations over the range of the index was similarly unlikely to correspond to meaningful changes in health risks (Capone et al., 2019). A study in a comparable setting in urban Harare likewise found little correspondence between household characteristics and *E. coli* contamination of multiple environmental reservoirs (Navab-Daneshmand et al., 2018). However, animal ownership in Harare was associated with higher soil contamination, a relationship also observed in rural Bangladesh but unexpectedly absent in our study (Ercumen et al., 2017b). Notably, all households in this study, the Harare study, and a small study in peri-urban Tanzania (Pickering et al., 2012) had some form of sanitation onsite, but measures of latrine quality were not associated with domestic fecal contamination. Some large studies in rural settings have found lower ambient fecal contamination in households with latrines (Boehm et al., 2016; Ercumen et al., 2017b) while other studies in similar settings did not (Odagiri et al., 2016), but associations between household latrine quality and fecal contamination have likewise not been observed (Ercumen et al., 2018b, 2017b). The presence and condition of shared or public toilets were also unrelated to FIB and enteric pathogens in urban public spaces in Kenya (Baker et al., 2018). In all cases, levels of fecal contamination were high throughout the study populations regardless of the sanitation technologies in use. While water-related infrastructure and practices, particularly on-premise piped water, have consistently shown fecal indicator reductions, these effects were restricted to transmission pathways directly tied to water usage and did not impact more distal pathways through the ambient environment (Ercumen et al., 2018a, 2018b; Navab-Daneshmand et al., 2018; Pickering et al., 2019a). However, there is some evidence that water infrastructure can indirectly support improvements to child health otherwise attributable to household sanitation (Reese et al., 2019), and reduced fecal indicator concentrations in drinking water have often, but not always, been associated reduced diarrhea risk (Ercumen et al., 2017a; Luby et al., 2015; Pickering et al., 2019a, 2018).

Despite analyzing more than 350 samples, by separately considering each of the five sample types the number of observations involved in any particular comparison was necessarily limited. As such, the power to identify risk factors among the variables considered was restricted for all but the largest effects—although only the largest of effects are likely to be meaningful for health, given the degree of fecal contamination observed. Furthermore, effect estimates were often unstable when both the response and risk factor variables were dichotomous, resulting in few or no observations for some combinations of response and risk factor values. Accordingly, care must be taken both to dismiss characteristics as potential risk factors when no associations were found and to identify a characteristic as a risk factor on the apparent strength of its association, which was likely highly sensitive to the particular set of data observed. Nevertheless, we did not find evidence of consistent relationships between fecal contamination and household characteristics. While the variables considered may be related to fecal contamination in the absolute sense, the range of conditions present in the study population may be too narrow to observe meaningful differences in contamination.

Our results show high levels of fecal contamination present in households sharing poor-quality sanitation facilities. Forthcoming results will include repeated measures of environmental samples following a sanitation intervention to better understand effects of sanitation improvements (Brown et al., 2015). Nevertheless, the broad distribution of microbial targets we observed, largely disconnected from variation in socioeconomic and sanitary characteristics, underscores the challenges to mitigating domestic fecal contamination through modifying conditions at the household level. Given the results of recent WaSH trials conducted in less populated settings, it is likely that transformational changes to the community environment are required before meaningful impacts on fecal contamination can be consistently realized (Cumming et al., 2019; Husseini et al., 2018; Pickering et al., 2019b). While fecal indicators are useful for identifying contaminated locations and MST approaches have proven valuable for drawing attention to underappreciated fecal sources, domestic animals and child feces in particular (Bauza et al., 2019; Harris et al., 2016), ambient fecal indicator measurements largely serve to confirm the pervasive nature of fecal contamination in settings with high burdens of enteric disease. Future research could benefit from directly assessing enteric pathogens in the environment and their relationships with fecal indicators, which has been rendered increasingly feasible by the recent development of multi-target quantitative molecular arrays and may provide clearer, more health-relevant signals for characterizing domestic fecal contamination (Baker et al., 2018; Fuhrmeister et al., 2019).

## References

[bib1] Baker K.K., O'Reilly C.E., Levine M.M., Kotloff K.L., Nataro J.P., Ayers T.L., Farag T.H., Nasrin D., Blackwelder W.C., Wu Y., Alonso P.L., Breiman R.F., Omore R., Faruque A.S.G., Das S.K., Ahmed S., Saha D., Sow S.O., Sur D., Zaidi A.K.M., Quadri F., Mintz E.D. (2016). Sanitation and hygiene-specific risk factors for moderate-to-severe diarrhea in young children in the global enteric multicenter study, 2007–2011: case-control study. PLoS Med..

[bib2] Baker K.K., Senesac R., Sewell D., Sen Gupta A., Cumming O., Mumma J. (2018). Fecal fingerprints of enteric pathogen contamination in public environments of Kisumu, Kenya, associated with human sanitation conditions and domestic animals. Environ. Sci. Technol..

[bib3] Barnes A.N., Mumma J., Cumming O. (2018). Role, ownership and presence of domestic animals in peri-urban households of Kisumu, Kenya. Zoonoses Public Health.

[bib4] Bauza V., Madadi V.O., Ocharo R.M., Nguyen T.H., Guest J.S. (2019). Microbial source tracking using 16S rRNA amplicon sequencing identifies evidence of widespread contamination from young children's feces in an urban slum of Nairobi, Kenya. Environ. Sci. Technol..

[bib5] Bauza V., Ocharo R.M., Nguyen T.H., Guest J.S. (2017). Soil ingestion is associated with child diarrhea in an urban slum of Nairobi, Kenya. Am. J. Trop. Med. Hyg..

[bib6] Berendes D.M., Kirby A.E., Clennon J.A., Agbemabiese C., Ampofo J.A., Armah G.E., Baker K.K., Liu P., Reese H.E., Robb K.A., Wellington N., Yakubu H., Moe C.L. (2018). Urban sanitation coverage and environmental fecal contamination: links between the household and public environments of Accra, Ghana. PloS One.

[bib7] Blackett I., Hawkins P., Heymans C. (2014). The Missing Link in Sanitation Service Delivery: a Review of Fecal Sludge Management in 12 Cities.

[bib8] Boehm A.B., Van De Werfhorst L.C., Griffith J.F., Holden P.A., Jay J.A., Shanks O.C., Wang D., Weisberg S.B. (2013). Performance of forty-one microbial source tracking methods: a twenty-seven lab evaluation study. Water Res..

[bib9] Boehm A.B., Wang D., Ercumen A., Shea M., Harris A.R., Shanks O.C., Kelty C., Ahmed A., Mahmud Z.H., Arnold B.F., Chase C., Kullmann C., Colford J.M., Luby S.P., Pickering A.J. (2016). Occurrence of host-associated fecal markers on child hands, household soil, and drinking water in rural Bangladeshi households. Environ. Sci. Technol. Lett. acs.estlett.6b00382.

[bib10] Briscoe J. (1984). Intervention studies and the definition of dominant transmission routes. Am. J. Epidemiol..

[bib11] Brown J., Cumming O., Bartram J., Cairncross S., Ensink J., Holcomb D., Knee J., Kolsky P., Liang K., Liang S., Nala R., Norman G., Rheingans R., Stewart J., Zavale O., Zuin V., Schmidt W.-P. (2015). A controlled, before-and-after trial of an urban sanitation intervention to reduce enteric infections in children: research protocol for the Maputo Sanitation (MapSan) study, Mozambique. BMJ Open.

[bib12] Bürkner P.-C. (2018). Advanced Bayesian multilevel modeling with the R package brms. R J..

[bib13] Bürkner P.-C. (2017). Brms : an R package for Bayesian multilevel models using stan. J. Stat. Software.

[bib14] Buuren S. van, Groothuis-Oudshoorn K. (2011). Mice : multivariate imputation by chained equations in R. J. Stat. Software.

[bib15] Byappanahalli M., Fujioka R. (2004). Indigenous soil bacteria and low moisture may limit but allow faecal bacteria to multiply and become a minor population in tropical soils. Water Sci. Technol..

[bib16] Cameron L.A., Shah M., Olivia S. (2013). Impact Evaluation of a Large-Scale Rural Sanitation Project in Indonesia.

[bib17] Cao Y., Griffith J.F., Dorevitch S., Weisberg S.B. (2012). Effectiveness of qPCR permutations, internal controls and dilution as means for minimizing the impact of inhibition while measuring Enterococcus in environmental waters. J. Appl. Microbiol..

[bib18] Capone D., Adriano Z., Berendes D., Cumming O., Dreibelbis R., Holcomb D.A., Knee J., Ross I., Brown J. (2019). A localized sanitation status index as a proxy for fecal contamination in urban Maputo, Mozambique. PloS One.

[bib19] Chern E.C., Siefring S., Paar J., Doolittle M., Haugland R.A. (2011). Comparison of quantitative PCR assays for Escherichia coli targeting ribosomal RNA and single copy genes. Lett. Appl. Microbiol..

[bib20] Clasen T., Boisson S., Routray P., Torondel B., Bell M., Cumming O., Ensink J., Freeman M., Jenkins M., Odagiri M., Ray S., Sinha A., Suar M., Schmidt W.-P. (2014). Effectiveness of a rural sanitation programme on diarrhoea, soil-transmitted helminth infection, and child malnutrition in Odisha, India: a cluster-randomised trial. Lancet Glob. Heal..

[bib21] Cox A.M., Goodwin K.D. (2013). Sample preparation methods for quantitative detection of DNA by molecular assays and marine biosensors. Mar. Pollut. Bull..

[bib22] Cumming O., Arnold B.F., Ban R., Clasen T., Esteves Mills J., Freeman M.C., Gordon B., Guiteras R., Howard G., Hunter P.R., Johnston R.B., Pickering A.J., Prendergast A.J., Prüss-Ustün A., Rosenboom J.W., Spears D., Sundberg S., Wolf J., Null C., Luby S.P., Humphrey J.H., Colford J.M. (2019). The implications of three major new trials for the effect of water, sanitation and hygiene on childhood diarrhea and stunting: a consensus statement. BMC Med..

[bib23] Delahoy M.J., Wodnik B., McAliley L., Penakalapati G., Swarthout J., Freeman M.C., Levy K. (2018). Pathogens transmitted in animal feces in low- and middle-income countries. Int. J. Hyg Environ. Health.

[bib24] Delignette-Muller M.L., Dutang C. (2015). Fitdistrplus: an R package for fitting distributions. J. Stat. Software.

[bib25] Eichmiller J.J., Miller L.M., Sorensen P.W. (2016). Optimizing techniques to capture and extract environmental DNA for detection and quantification of fish. Mol. Ecol. Resour..

[bib26] Ercumen A., Arnold B.F., Naser A.M., Unicomb L., Colford J.M., Luby S.P. (2017). Potential sources of bias in the use of Escherichia coli to measure waterborne diarrhoea risk in low-income settings. Trop. Med. Int. Health.

[bib27] Ercumen A., Mertens A., Arnold B.F., Benjamin-Chung J., Hubbard A.E., Ahmed M.A., Kabir M.H., Rahman Khalil M.M., Kumar A., Rahman M.S., Parvez S.M., Unicomb L., Rahman M., Ram P.K., Clasen T., Luby S.P., Colford J.M. (2018). Effects of single and combined water, sanitation and handwashing interventions on fecal contamination in the domestic environment: a cluster-randomized controlled trial in rural Bangladesh. Environ. Sci. Technol..

[bib28] Ercumen A., Pickering A.J., Kwong L.H., Arnold B.F., Parvez S.M., Alam M., Sen D., Islam S., Kullmann C., Chase C., Ahmed R., Unicomb L., Luby S.P., Colford J.M. (2017). Animal feces contribute to domestic fecal contamination: evidence from E. coli measured in water, hands, food, flies, and soil in Bangladesh. Environ. Sci. Technol..

[bib29] Ercumen A., Pickering A.J., Kwong L.H., Mertens A., Arnold B.F., Benjamin-Chung J., Hubbard A.E., Alam M., Sen D., Islam S., Rahman M.Z., Kullmann C., Chase C., Ahmed R., Parvez S.M., Unicomb L., Rahman M., Ram P.K., Clasen T., Luby S.P., Colford J.M. (2018). Do sanitation improvements reduce fecal contamination of water, hands, food, soil, and flies? Evidence from a cluster-randomized controlled trial in rural Bangladesh. Environ. Sci. Technol..

[bib30] Ezeh A., Oyebode O., Satterthwaite D., Chen Y.-F.F., Ndugwa R., Sartori J., Mberu B., Melendez-Torres G.J., Haregu T., Watson S.I., Caiaffa W., Capon A., Lilford R.J. (2017). The history, geography, and sociology of slums and the health problems of people who live in slums. Lancet.

[bib31] Field K.G., Samadpour M. (2007). Fecal source tracking, the indicator paradigm, and managing water quality. Water Res..

[bib32] Finch J.E., Prince J., Hawksworth M. (1978). A bacteriological survey of the domestic environment. J. Appl. Bacteriol..

[bib33] Fluss R., Faraggi D., Reiser B. (2005). Estimation of the youden index and its associated cutoff point. Biom. J..

[bib34] Fuhrmeister E.R., Ercumen A., Pickering A.J., Jeanis K.M., Ahmed M., Brown S., Arnold B.F., Hubbard A.E., Alam M., Sen D., Islam S., Kabir M.H., Kwong L.H., Islam M., Unicomb L., Rahman M., Boehm A.B., Luby S.P., Colford J.M., Nelson K.L. (2019). Predictors of enteric pathogens in the domestic environment from human and animal sources in rural Bangladesh. Environ. Sci. Technol..

[bib35] Gelman A., Hill J. (2007). Data Analysis Using Regression and Multilevel/hierarchical Models.

[bib36] Gelman A., Hill J., Yajima M. (2012). Why we (usually) don't have to worry about multiple comparisons. J. Res. Educ. Eff..

[bib37] Gelman A., Jakulin A., Pittau M.G., Su Y.-S. (2008). A weakly informative default prior distribution for logistic and other regression models. Ann. Appl. Stat..

[bib38] Gentry-Shields J., Rowny J.G., Stewart J.R. (2012). HuBac and nifH source tracking markers display a relationship to land use but not rainfall. Water Res..

[bib39] Green H.C., Dick L.K., Gilpin B., Samadpour M., Field K.G. (2012). Genetic markers for rapid PCR-based identification of gull, Canada goose, duck, and chicken fecal contamination in water. Appl. Environ. Microbiol..

[bib40] Green H.C., Haugland R.A., Varma M., Millen H.T., Borchardt M.A., Field K.G., Walters W.A., Knight R., Sivaganesan M., Kelty C.A., Shanks O.C. (2014). Improved HF183 quantitative real-time PCR assay for characterization of human fecal pollution in ambient surface water samples. Appl. Environ. Microbiol..

[bib41] Griffith J.F., Layton B.a., Boehm A.B., Holden P.a., Jay J.a., Hagedorn C., McGee C.D., Weisberg S.B. (2013). The California Microbial Source Identification Manual: A Tiered Approach to Identifying Fecal Pollution Sources to Beaches.

[bib42] Gruber J.S., Ercumen A., Colford J.M. (2014). Coliform bacteria as indicators of diarrheal risk in household drinking water: systematic review and meta-analysis. PloS One.

[bib43] Harmel D., Wagner K., Martin E., Smith D., Wanjugi P., Gentry T., Gregory L., Hendon T. (2016). Effects of field storage method on E. coli concentrations measured in storm water runoff. Environ. Monit. Assess..

[bib44] Harris A.R., Davis J., Boehm A.B. (2013). Mechanisms of post-supply contamination of drinking water in Bagamoyo, Tanzania. J. Water Health.

[bib45] Harris A.R., Pickering A.J., Boehm A.B., Mrisho M., Davis J. (2019). Comparison of analytical techniques to explain variability in stored drinking water quality and microbial hand contamination of female caregivers in Tanzania. Environ. Sci. Process. Impacts.

[bib46] Harris A.R., Pickering A.J., Harris M., Doza S., Islam M.S., Unicomb L., Luby S., Davis J., Boehm A.B. (2016). Ruminants contribute fecal contamination to the urban household environment in Dhaka, Bangladesh. Environ. Sci. Technol..

[bib47] Harwood V.J., Boehm A.B., Sassoubre L.M., Vijayavel K., Stewart J.R., Fong T.T., Caprais M.P., Converse R.R., Diston D., Ebdon J., Fuhrman J.A., Gourmelon M., Gentry-Shields J., Griffith J.F., Kashian D.R., Noble R.T., Taylor H., Wicki M. (2013). Performance of viruses and bacteriophages for fecal source determination in a multi-laboratory, comparative study. Water Res..

[bib48] Harwood V.J., Staley C., Badgley B.D., Borges K., Korajkic A. (2014). Microbial source tracking markers for detection of fecal contamination in environmental waters: relationships between pathogens and human health outcomes. FEMS Microbiol. Rev..

[bib49] Haugland R. a, Siefring S.C., Wymer L.J., Brenner K.P., Dufour A.P. (2005). Comparison of Enterococcus measurements in freshwater at two recreational beaches by quantitative polymerase chain reaction and membrane filter culture analysis. Water Res..

[bib50] Haugland R.A., Siefring S., Lavender J., Varma M. (2012). Influences of sample interference and interference controls on quantification of enterococci fecal indicator bacteria in surface water samples by the qPCR method. Water Res..

[bib51] Hawkins P., Blackett I., Heymans C. (2013). Poor-inclusive Urban Sanitation: an Overview.

[bib52] Hedin G., Rynbäck J., Loré B. (2010). New technique to take samples from environmental surfaces using flocked nylon swabs. J. Hosp. Infect..

[bib53] Heijnen M., Routray P., Torondel B., Clasen T. (2015). Neighbour-shared versus communal latrines in urban slums: a cross-sectional study in Orissa, India exploring household demographics, accessibility, privacy, use and cleanliness. Trans. R. Soc. Trop. Med. Hyg..

[bib54] Holcomb D.A., Messier K.P., Serre M.L., Rowny J.G., Stewart J.R. (2018). Geostatistical prediction of microbial water quality throughout a stream network using meteorology, land cover, and spatiotemporal autocorrelation. Environ. Sci. Technol..

[bib55] Humphrey J.H., Mbuya M.N.N., Ntozini R., Moulton L.H., Stoltzfus R.J., Tavengwa N.V., Mutasa K., Majo F., Mutasa B., Mangwadu G., Chasokela C.M., Chigumira A., Chasekwa B., Smith L.E., Tielsch J.M., Jones A.D., Manges A.R., Maluccio J.A., Prendergast A.J. (2019). Independent and combined effects of improved water, sanitation, and hygiene, and improved complementary feeding, on child stunting and anaemia in rural Zimbabwe: a cluster-randomised trial. Lancet Glob. Heal..

[bib56] Husseini M., Darboe M.K., Moore S.E., Nabwera H.M., Prentice A.M. (2018). Thresholds of socio-economic and environmental conditions necessary to escape from childhood malnutrition: a natural experiment in rural Gambia. BMC Med..

[bib57] Jenkins M.W., Tiwari S., Lorente M., Gichaba C.M., Wuertz S. (2009). Identifying human and livestock sources of fecal contamination in Kenya with host-specific Bacteroidales assays. Water Res..

[bib58] Johnston C., Byappanahalli M.N., Gibson J.M., Ufnar J.a., Whitman R.L., Stewart J.R. (2013). Probabilistic analysis showing that a combination of Bacteroides and methanobrevibacter source tracking markers is effective for identifying waters contaminated by human fecal pollution. Environ. Sci. Technol..

[bib59] Johnston C., Ufnar J. a, Griffith J.F., Gooch J. a, Stewart J.R. (2010). A real-time qPCR assay for the detection of the nifH gene of Methanobrevibacter smithii, a potential indicator of sewage pollution. J. Appl. Microbiol..

[bib60] Jothikumar N., Cromeans T.L., Vincent R., Lu X., Sobsey M.D., Erdman D.D., Hill V.R. (2005). Quantitative real-time PCR assays for detection of human adenoviruses and identification of serotypes 40 and 41 quantitative real-time PCR assays for detection of human adenoviruses and identification of serotypes 40 and 41. Appl. Environ. Microbiol..

[bib61] Julian T.R. (2016). Environmental transmission of diarrheal pathogens in low and middle income countries. Environ. Sci. Process. Impacts.

[bib62] Kildare B.J., Leutenegger C.M., McSwain B.S., Bambic D.G., Rajal V.B., Wuertz S. (2007). 16S rRNA-based assays for quantitative detection of universal, human-, cow-, and dog-specific fecal Bacteroidales: a Bayesian approach. Water Res..

[bib63] Knee J., Sumner T., Adriano Z., Berendes D., de Bruijn E., Schmidt W., Nalá R., Cumming O., Brown J. (2018). Risk factors for childhood enteric infection in urban Maputo, Mozambique: a cross-sectional study. PLoS Neglected Trop. Dis..

[bib64] Korajkic A., McMinn B., Harwood V. (2018). Relationships between microbial indicators and pathogens in recreational water settings. Int. J. Environ. Res. Publ. Health.

[bib65] Layton B. a, Cao Y., Ebentier D.L., Hanley K., Ballesté E., Brandão J., Byappanahalli M., Converse R., Farnleitner A.H., Gentry-Shields J., Gidley M.L., Gourmelon M., Lee C.S., Lee J., Lozach S., Madi T., Meijer W.G., Noble R., Peed L., Reischer G.H., Rodrigues R., Rose J.B., Schriewer A., Sinigalliano C., Srinivasan S., Stewart J., Van De Werfhorst L.C., Wang D., Whitman R., Wuertz S., Jay J., Holden P. a, Boehm A.B., Shanks O., Griffith J.F. (2013). Performance of human fecal anaerobe-associated PCR-based assays in a multi-laboratory method evaluation study. Water Res..

[bib66] Levy K., Nelson K.L., Hubbard A., Eisenberg J.N.S. (2012). Rethinking indicators of microbial drinking water quality for health studies in tropical developing countries: case study in northern coastal Ecuador. Am. J. Trop. Med. Hyg..

[bib67] Lion T. (2014). Adenovirus infections in immunocompetent and immunocompromised patients. Clin. Microbiol. Rev..

[bib68] Luby S.P., Halder A.K., Huda T.M., Unicomb L., Islam M.S., Arnold B.F., Johnston R.B. (2015). Microbiological contamination of drinking water associated with subsequent child diarrhea. Am. J. Trop. Med. Hyg..

[bib69] Luby S.P., Rahman M., Arnold B.F., Unicomb L., Ashraf S., Winch P.J., Stewart C.P., Begum F., Hussain F., Benjamin-Chung J., Leontsini E., Naser A.M., Parvez S.M., Hubbard A.E., Lin A., Nizame F.A., Jannat K., Ercumen A., Ram P.K., Das K.K., Abedin J., Clasen T.F., Dewey K.G., Fernald L.C., Null C., Ahmed T., Colford J.M. (2018). Effects of water quality, sanitation, handwashing, and nutritional interventions on diarrhoea and child growth in rural Bangladesh: a cluster randomised controlled trial. Lancet Glob. Heal..

[bib70] Mahmoudi N., Slater G.F., Fulthorpe R.R. (2011). Comparison of commercial DNA extraction kits for isolation and purification of bacterial and eukaryotic DNA from PAH-contaminated soils. Can. J. Microbiol..

[bib71] Mara D., Lane J., Scott B., Trouba D. (2010). Sanitation and health. PLoS Med..

[bib72] Mayer R.E., Reischer G.H., Ixenmaier S.K., Derx J., Blaschke A.P., Ebdon J.E., Linke R., Egle L., Ahmed W., Blanch A.R., Byamukama D., Savill M., Mushi D., Cristóbal H.A., Edge T.A., Schade M.A., Aslan A., Brooks Y.M., Sommer R., Masago Y., Sato M.I., Taylor H.D., Rose J.B., Wuertz S., Shanks O.C., Piringer H., Mach R.L., Savio D., Zessner M., Farnleitner A.H. (2018). Global distribution of human-associated fecal genetic markers in reference samples from six continents. Environ. Sci. Technol..

[bib73] McElreath R. (2015). Statistical Rethinking: A Bayesian Course with Examples in R and Stan.

[bib74] McGranahan G. (2015). Realizing the right to sanitation in deprived urban communities: meeting the challenges of collective action, coproduction, affordability, and housing tenure. World Dev..

[bib75] McLellan S.L., Eren A.M. (2014). Discovering new indicators of fecal pollution. Trends Microbiol..

[bib76] Mersmann O., Trautmann H., Steuer D., Bornkamp B. (2018). Truncnorm.

[bib77] Messier K.P., Akita Y., Serre M.L. (2012). Integrating address geocoding, land use regression, and spatiotemporal geostatistical estimation for groundwater tetrachloroethylene. Environ. Sci. Technol..

[bib78] Moore G., Griffith C. (2007). Problems associated with traditional hygiene swabbing: the need for in-house standardization. J. Appl. Microbiol..

[bib79] Navab-Daneshmand T., Friedrich M.N.D., Gächter M., Montealegre M.C., Mlambo L.S., Nhiwatiwa T., Mosler H.-J., Julian T.R. (2018). Escherichia coli contamination across multiple environmental compartments (soil, hands, drinking water, and handwashing water) in urban Harare: correlations and risk factors. Am. J. Trop. Med. Hyg..

[bib80] Null C., Stewart C.P., Pickering A.J., Dentz H.N., Arnold B.F., Arnold C.D., Benjamin-Chung J., Clasen T., Dewey K.G., Fernald L.C.H., Hubbard A.E., Kariger P., Lin A., Luby S.P., Mertens A., Njenga S.M., Nyambane G., Ram P.K., Colford J.M. (2018). Effects of water quality, sanitation, handwashing, and nutritional interventions on diarrhoea and child growth in rural Kenya: a cluster-randomised controlled trial. Lancet Glob. Heal..

[bib81] Nutz S., Döll K., Karlovsky P. (2011). Determination of the LOQ in real-time PCR by receiver operating characteristic curve analysis: application to qPCR assays for Fusarium verticillioides and F. proliferatum. Anal. Bioanal. Chem..

[bib82] Odagiri M., Schriewer A., Daniels M.E., Wuertz S., Smith W.A., Clasen T., Schmidt W., Jin Y., Torondel B., Misra P.R., Panigrahi P., Jenkins M.W. (2016). Human fecal and pathogen exposure pathways in rural Indian villages and the effect of increased latrine coverage. Water Res..

[bib83] Odagiri M., Schriewer A., Hanley K., Wuertz S., Misra P.R., Panigrahi P., Jenkins M.W. (2015). Validation of Bacteroidales quantitative PCR assays targeting human and animal fecal contamination in the public and domestic domains in India. Sci. Total Environ..

[bib84] Oh S., Buddenborg S., Yoder-Himes D.R., Tiedje J.M., Konstantinidis K.T. (2012). Genomic diversity of Escherichia isolates from Diverse habitats. PloS One.

[bib85] Patil S.R., Arnold B.F., Salvatore A.L., Briceno B., Ganguly S., Colford J.M., Gertler P.J., Colford J.M., Gertler P.J. (2014). The effect of India's total sanitation campaign on defecation behaviors and child health in rural Madhya Pradesh: a cluster randomized controlled trial. PLoS Med..

[bib86] Penakalapati G., Swarthout J., Delahoy M.J., McAliley L., Wodnik B., Levy K., Freeman M.C. (2017). Exposure to animal feces and human health: a systematic review and proposed research priorities. Environ. Sci. Technol..

[bib87] Pickering A.J., Crider Y., Sultana S., Swarthout J., Goddard F.G., Anjerul Islam S., Sen S., Ayyagari R., Luby S.P. (2019). Effect of in-line drinking water chlorination at the point of collection on child diarrhoea in urban Bangladesh: a double-blind, cluster-randomised controlled trial. Lancet Glob. Heal..

[bib88] Pickering A.J., Djebbari H., Lopez C., Coulibaly M., Alzua M.L. (2015). Effect of a community-led sanitation intervention on child diarrhoea and child growth in rural Mali: a cluster-randomised controlled trial. Lancet Glob. Heal..

[bib89] Pickering A.J., Ercumen A., Arnold B.F., Kwong L.H., Parvez S.M., Alam M., Sen D., Islam S., Kullmann C., Chase C., Ahmed R., Unicomb L., Colford J.M., Luby S.P. (2018). Fecal indicator bacteria along multiple environmental transmission pathways (water, hands, food, soil, flies) and subsequent child diarrhea in rural Bangladesh. Environ. Sci. Technol..

[bib90] Pickering A.J., Julian T.R., Marks S.J., Mattioli M.C., Boehm A.B., Schwab K.J., Davis J. (2012). Fecal contamination and diarrheal pathogens on surfaces and in soils among Tanzanian households with and without improved sanitation. Environ. Sci. Technol..

[bib91] Pickering A.J., Null C., Winch P.J., Mangwadu G., Arnold B.F., Prendergast A.J., Njenga S.M., Rahman M., Ntozini R., Benjamin-Chung J., Stewart C.P., Huda T.M.N., Moulton L.H., Colford J.M., Luby S.P., Humphrey J.H. (2019). The WASH Benefits and SHINE trials: interpretation of WASH intervention effects on linear growth and diarrhoea. Lancet Glob. Heal..

[bib92] Pontiroli A., Travis E.R., Sweeney F.P., Porter D., Gaze W.H., Mason S., Hibberd V., Holden J., Courtenay O., Wellington E.M.H. (2011). Pathogen quantitation in complex matrices: a multi-operator comparison of DNA extraction methods with a novel assessment of PCR inhibition. PloS One.

[bib93] Pope M.L., Bussen M., Feige M.A., Shadix L., Gonder S., Rodgers C., Chambers Y., Pulz J., Miller K., Connell K., Standridge J. (2003). Assessment of the effects of holding time and temperature on Escherichia coli densities in surface water samples. Appl. Environ. Microbiol..

[bib94] Prendergast A.J., Gharpure R., Mor S., Viney M., Dube K., Lello J., Berger C., Siwila J., Joyeux M., Hodobo T., Hurt L., Brown T., Hoto P., Tavengwa N., Mutasa K., Craddock S., Chasekwa B., Robertson R.C., Evans C., Chidhanguro D., Mutasa B., Majo F., Smith L.E., Hirai M., Ntozini R., Humphrey J.H., Berendes D. (2019). Putting the “A” into WaSH: a call for integrated management of water, animals, sanitation, and hygiene. Lancet Planet. Heal..

[bib95] R Core Team (2018). R: A Language and Environment for Statistical Computing.

[bib96] Reese H., Sinharoy S.S., Clasen T. (2019). Using structural equation modelling to untangle sanitation, water and hygiene pathways for intervention improvements in height-for-age in children <5 years old. Int. J. Epidemiol..

[bib97] Reischer G.H., Ebdon J.E., Bauer J.M., Schuster N., Ahmed W., Aström J., Blanch A.R., Blöschl G., Byamukama D., Coakley T., Ferguson C., Goshu G., Ko G., de Roda Husman A.M., Mushi D., Poma R., Pradhan B., Rajal V., Schade M.a., Sommer R., Taylor H., Toth E.M., Vrajmasu V., Wuertz S., MacH R.L., Farnleitner A.H., Astrom J., Blanch A.R., Bloschl G., Byamukama D., Coakley T., Ferguson C., Goshu G., Ko G., de Roda Husman A.M., Mushi D., Poma R., Pradhan B., Rajal V., Schade M.a., Sommer R., Taylor H., Toth E.M., Vrajmasu V., Wuertz S., MacH R.L., Farnleitner A.H., Aström J., Blanch A.R., Blöschl G., Byamukama D., Coakley T., Ferguson C., Goshu G., Ko G., de Roda Husman A.M., Mushi D., Poma R., Pradhan B., Rajal V., Schade M.a., Sommer R., Taylor H., Toth E.M., Vrajmasu V., Wuertz S., MacH R.L., Farnleitner A.H. (2013). Performance characteristics of qPCR assays targeting human- and ruminant-associated bacteroidetes for microbial source tracking across sixteen countries on six continents. Environ. Sci. Technol..

[bib98] Rivera S.C., Tc H., Ga T. (1988). Isolation of fecal coliform from pristine sites in a tropical rain forest. Appl. Environ. Microbiol..

[bib99] Schriewer A., Odagiri M., Wuertz S., Misra P.R., Panigrahi P., Clasen T., Jenkins M.W. (2015). Human and animal fecal contamination of community water sources, stored drinking water and hands in rural India measured with validated microbial source tracking assays. Am. J. Trop. Med. Hyg..

[bib100] Sclar G.D., Penakalapati G., Amato H.K., Garn J.V., Alexander K., Freeman M.C., Boisson S., Medlicott K.O., Clasen T. (2016). Assessing the impact of sanitation on indicators of fecal exposure along principal transmission pathways: a systematic review. Int. J. Hyg Environ. Health.

[bib101] Scott E., Bloomfield S.F., Barlow C.G. (1982). An investigation of microbial contamination in the home. J. Hyg..

[bib102] Shiras T., Cumming O., Brown J., Muneme B., Nala R., Dreibelbis R. (2018). Shared latrines in Maputo, Mozambique: exploring emotional well-being and psychosocial stress. BMC Int. Health Hum. Right.

[bib103] Shiras T., Cumming O., Brown J., Muneme B., Nala R., Dreibelbis R., Shiras T., Cumming O., Brown J., Muneme B., Nala R., Dreibelbis R. (2018). Shared sanitation management and the role of social capital: findings from an urban sanitation intervention in Maputo, Mozambique. Int. J. Environ. Res. Publ. Health.

[bib104] Shrestha A., Dorevitch S. (2019). Evaluation of rapid qPCR method for quantification of E. coli at non-point source impacted Lake Michigan beaches. Water Res..

[bib105] Simiyu S., Swilling M., Cairncross S., Rheingans R. (2017). Determinants of quality of shared sanitation facilities in informal settlements: case study of Kisumu, Kenya. BMC Publ. Health.

[bib106] Sinharoy S.S., Pittluck R., Clasen T. (2019). Review of drivers and barriers of water and sanitation policies for urban informal settlements in low-income and middle-income countries. Util. Pol..

[bib107] Sinharoy S.S., Schmidt W.-P., Wendt R., Mfura L., Crossett E., Grépin K.A., Jack W., Rwabufigiri B.N., Habyarimana J., Clasen T. (2017). Effect of community health clubs on child diarrhoea in western Rwanda: cluster-randomised controlled trial. Lancet Glob. Heal..

[bib108] Sitoe A., Breiman R.F., Bassat Q. (2018). Child mortality in Mozambique: a review of recent trends and attributable causes. Curr. Trop. Med. Reports.

[bib109] Sivaganesan M., Haugland R.a., Chern E.C., Shanks O.C. (2010). Improved strategies and optimization of calibration models for real-time PCR absolute quantification. Water Res..

[bib110] Sivaganesan M., Siefring S., Varma M., Haugland R.A., Shanks O.C. (2008). A Bayesian method for calculating real-time quantitative PCR calibration curves using absolute DNA standards. BMC Bioinf..

[bib111] Solo-Gabriele H.M., Wolfert M.A., Desmarais T.R., Palmer C.J. (2000). Sources of Escherichia coli in a coastal subtropical environment. Appl. Environ. Microbiol..

[bib112] Stan Development Team (2019). Stan User's Guide.

[bib113] Stan Development Team (2019). Prior choice recommendations. https://github.com/stan-dev/stan/wiki/Prior-Choice-Recommendations.

[bib114] Stewart J.R., Boehm A.B., Dubinsky E.A., Fong T.-T., Goodwin K.D., Griffith J.F., Noble R.T., Shanks O.C., Vijayavel K., Weisberg S.B. (2013). Recommendations following a multi-laboratory comparison of microbial source tracking methods. Water Res..

[bib115] UN-HABITAT (2014). The State of African Cities: Re-imagining Sustainable Urban Transitions.

[bib116] USEPA (2009). Method 1603: Escherichia coli (E.Coli) in Water by Membrane Filtration Using Modified Membrane-Thermotolerant Escherichia coli Agar, EPA-821-R-09-007.

[bib117] Vujcic J., Ram P.K., Hussain F., Unicomb L., Gope P.S., Abedin J., Mahmud Z.H., Sirajul Islam M., Luby S.P. (2014). Toys and toilets: cross-sectional study using children's toys to evaluate environmental faecal contamination in rural Bangladeshi households with different sanitation facilities and practices. Trop. Med. Int. Health.

[bib118] Wagner E.G., Lanoix J.N. (1958). Excreta disposal for rural areas and small communities. Monogr. Ser. World Health Organ..

[bib119] Weidhaas J.L., Macbeth T.W., Olsen R.L., Sadowsky M.J., Norat D., Harwood V.J. (2010). Identification of a Brevibacterium marker gene specific to poultry litter and development of a quantitative PCR assay. J. Appl. Microbiol..

[bib120] WHO/UNICEF (2019). Progress on Household Drinking Water, Sanitation and Hygiene 2000-2017: Special Focus on Inequalities.

[bib121] WHO (1997). Guidelines for Drinking-Water Quality.

