# Supplementary material for: Human fecal contamination of water, soil, and surfaces in households sharing poor-quality sanitation facilities in Maputo, Mozambique
Source: Int J Hyg Environ Health. 2020 May;226:113496. doi: 10.1016/j.ijheh.2020.113496 (PMC7174141; doi:10.1016/j.ijheh.2020.113496)
Supplement: Multimedia component 2 [file mmc2.pdf]

Human Fecal Contamination of Water, Soil, and Surfaces in Households Sharing Poor-Quality Sanitation Facilities in Maputo, Mozambique: Supplementary Material

David A. Holcomb, Jackie Knee, Trent Sumner, Zaida Adriano, Ellen de Bruijn, Rassul Nalá, Oliver Cumming, Joe Brown, Jill R. Stewart

This supplementary material contains 21 pages and 7 tables.

Table of Contents

S1. Reference material for qPCR standard curves 3

S2. Fecal sludge sampling apparatus 6

S3. DNA isolation from fecal samples for MST assay validation 6

S4. Assay diagnostic performance calculations 7

S5. Processing of environmental samples 7

S6. Analysis of unextracted reference material 8

S7. Survival modeling to estimate detection limits and extraction efficiency 8

S8. Calibration curve and extraction efficiency estimates 9

S9. Variables assessed in risk factor analysis 11

S10. Tabular summaries of model results 15

S11. Supplementary References 20

S1. Reference material for qPCR standard curves

Nucleotide Basic Local Alignment Search Tool (BLAST) searches were performed with the published primers and probe sequences (Table S1) for each candidate assay to ensure published sequence accuracy and to obtain the expected amplicon sequence (Agarwala et al., 2016). The matching amplicon sequence and ten additional bases on both ends were extracted from the GenBank database to serve as reference sequences (Clark et al., 2016). Because all three assays targeting *Bacteroidales* 16S rRNA genes matched the same *B. dorei* gene sequence, a single reference sequence was extracted spanning the entire region targeted by these assays. The reference sequences obtained for the avian-associated assays were concatenated to construct a composite reference sequence for both assays. The reference sequences for the remaining (non-avian, non-16S) assays were likewise concatenated. These three composite reference sequences (Table S2) were commercially synthesized as gBlock artificial linear plasmids (Integrated DNA Technologies, Skokie, Il, USA) to serve as standard reference material for all candidate assays (Kodani and Winchell, 2012; Liu et al., 2013).

Table S1. qPCR assay details

assay	oligonucleotide (nM)	sequence (5'-3')	GenBank (base positions)	cycles	parameters	reference
BacUni-UCD	BacUni-520f (400)	CGTTATCCGGATTTATTGGGTTTA	AB242142 (544-567)	40	15 s: 95 °C<br>60 s: 60 °C	(Kildare et al., 2007)
	BacUni-690r1 (400)	CAATCGGAGTTCTTCGTGATATCTA	AB242142 (696-720)			
	BacUni-690r2 (400)	AATCGGAGTTCCTCGTGATATCTA	AB242142 (696-719)			
	BacUni-656p (80)	6-FAM-TGGTGTAGCGGTGAAA-BHQplus	AB242142 (676-691)			
EC23S857	F (1000)	GGTAGAGCACTGTTTtGGCA	DQ682619 (857-876)	40	15 s: 95 °C<br>60 s: 60 °C	(Chern et al., 2011)
	R (1000)	TGTCTCCCGTGATAACtTTCTC	DQ682619 (923-944)			
	P (80)	6-FAM-TCATCCCGACTTACCAACCCG-BHQ1	DQ682619 (883-903)			
BacHum-UCD	BacHum-160f (400)	TGAGTTCACATGTCCGCATGA	AB242142 (184-204)	40	15 s: 95 °C<br>60 s: 60 °C	(Kildare et al., 2007)
	BacHum-241r (400)	CGTTACCCCGCCTACTATCTAATG	AB242142 (242-265)			
	BacHum-193p (80)	6-FAM-TCCGGTAGACGATGGGGATGCGTT-BHQ1	AB242142 (217-240)			
HAdV	JTVXF (250)	GGACGCCTCGGAGTACCTGAG	AC_000008 (18895-18915)	45	10 s: 95 °C<br>30 s: 55 °C<br>15 s: 72 °C	(Jothikumar et al., 2005)
	JTVXR (250)	ACIGTGGGGTTTCTGAACTTGTT	AC_000008 (18968-18990)			
	JTVXP (150)	6-FAM-CTGGTGCAGTTCGCCCGTGCCA-BHQ1	AC_000008 (18923-18944)			
HF183/BacR287	HF183 (1000)	ATCATGAGTTCACATGTCCG	AB242142 (180-199)	40	15 s: 95 °C<br>60 s: 60 °C	(Green et al., 2014)
	BacR287 (1000)	CTTCCTCTCAGAACCCCTATCC	AB242142 (284-305)			
	BacP234MGB (80)	6-FAM-CTAATGGAACGCATCCC-BHQplus	AB242142 (231-247)			
Mnif	Mnif-202F (800)	GAAAGCGGAGGTCCTGAA	AB019138 (202-219)	50	10 s: 95 °C<br>30 s: 57 °C	(Johnston et al., 2010)
	Mnif-353R (800)	ACTGAAAAACCTCCGCAAAC	AB019138 (334-353)			
	Mnif-236P (240)	6-FAM-CCGGACGTGGTGTAACAGTAGCTA-BHQ1	AB019138 (236-259)			
GFD	F (100)	TcGGCTGAGCACTCTAGGG	JN084061 (39-57)	40	15 s: 95 °C<br>32 s: 57 °C	(Green et al., 2012)
	R (100)	GCGTCTCTTTGTACATCCCA	JN084061 (142-161)			
LA35	LA35F (500)	ACCGGATACGACCATCTGC	FJ462358 (166-184)	45	15 s: 95 °C<br>30 s: 60 °C<br>30 s: 72 °C	(Weidhaas et al., 2010)
	LA35R (500)	TCCCCAGTGTCAGTCACAGC	FJ462358 (717-736)			
Sketa22	SketaF2 (1000)	GGTTTCCGCAGCTGGG	AF170538 (22-37)	40	15 s: 95 °C<br>60 s: 60 °C	(Haugland et al., 2010, 2005)
	SketaR22 (1000)	CCGAGCCGTCCTGGTC	AF170538 (83-98)			
	SketaP2 (80)	6-FAM-AGTCGCAGGCGGCCACCGT-BHQ1	AF170538 (40-58)]			

Table S2. Composite standard reference material for qPCR assays

standard	sequence (5'-3')	GenBank<br>(base positions)	length<br>(bases)	assays covered
bdorei	CCAGGATGGGATCATGAGTTCACATGTCCGCATGATTAAAGGTATTTTCCGGTA<br>GACGATGGGGATGCGTTCCATTAGATAGTAGGCGGGGTAACGGCCCACCTAGT<br>CAACGATGGATAGGGGTTCTGAGAGGAAGGTCCCCCACATTGGAACTGAGACA<br>CGGTCCAAACTCCTACGGGAGGCAGCAGTGAGGAATATTGGTCAATGGGCGAT<br>GGCCTGAACCAGCCAAGTAGCGTGAAGGATGACTGCCCTATGGGTTGTAAACT<br>TCTTTTATAAAGGAATAAAGTCGGGTATGCATACCCGTTTGCATGTACTTTATG<br>AATAAGGATCGGCTAACTCCGTGCCAGCAGCCGCGGTAATACGGAGGATCCGA<br>GCGTTATCCGGATTTATTGGGTTTAAAGGGAGCGTAGATGGATGTTTAAGTCA<br>GTTGTGAAAGTTTGCGGCTCAACCGTAAAATTGCAGTTGATACTGGATGTCTTG<br>AGTGCAGTTGAGGCAGGCGGAATTCGTGGTGTAGCGGTGAAATGCTTAGATAT<br>CACGAAGAACTCCGATTGCGAAGGCAGC	AB242142 (170-730)	560	BacHum-UCD<br>BacUni-UCD<br>HF183/BacR287
bird	TGGGTCTAATACCGGATACGACCATCTGCCGCATGGCGGGTGGTGGAAAGTTT<br>TTCGATTGGGGATGGGCTCGCGGCCTATCAGTTTGTTGGTGGGGTAATGGCCTA<br>CCAAGGCGACGACGGGTAGCCGGCCTGAGAGGGCGACCGGCCACACTGGGAC<br>TGAGACACGGCCCAGACTCCTACGGGAGGCAGCAGTGGGGAATATTGCACAAT<br>GGGGGAAACCCTGATGCAGCGACGCAGCGTGCGGGATGACGGCCTTCGGGTTG<br>TAAACCGCTTTCAGCAGGGAAGAAGCCTTCGGGTGACGGTACCTGCAGAAGAA<br>GTACCGGCTAACTACGTGCCAGCAGCCGCGGTAATACGTAGGGTACGAGCGTT<br>GTCCGGAATTATTGGGCGTAAAGAGCTCGTAGGTGGTTGGTCACGTCTGCTGT<br>GGAAACGCAACGCTTAACGTTGCGCGGGCAGTGGGTACGGGCTGACTAGAGTG<br>CAGTAGGGGAGTCTGGAATTCCTGGTGTAGCGGTGAAATGCGCAGATATCAGG<br>AGGAACACCGGTGGCGAAGGCGGGACTCTGGGCTGTGACTGACACTGGGGAG<br>CGAAAGCATTGCTAACAGTTcGGCTGAGCACTCTAGGGAGACTGCCTTCGCAA<br>GGAGGAGGAAGGTGAGGACGACGTCAAGTCATCATGGCCCTTACGCCTAGGG<br>CTACACACGTGCTACAATGGGATGTACAAAGAGACGCAATACCGCGA	FJ462358 (156-746)<br>JN084061 (29-171)	732	GFD<br>LA35
non16S	TAACTATGGTCATCGTTCGTCAGCAGTAACAGTAATTGCTACACCTGCTGAAAC<br>CACTGTCCCTTTTTCTTGGGCAACTCTTGTTTATGTGTTGAAAGCGGAGGTCCT<br>GAACCGGGTGTTGGCTGTGCCGGACGTGGTGTAACAGTAGCTATGAAAAGACT<br>TGAAAACTTAGGTGTTTTTGATAAGGATTTGGATGTAGTCATTTATGGTGTACT<br>TGGAGATGTTGTTTGCGGAGGTTTTTCAGTGCCTTTACGTTCTCGGGCCAGGAC<br>GCCTCGGAGTACCTGAGCCCCGGGCTGGTGCAGTTTGCCCGCGCCACCGAGAC<br>GTACTTCAGCCTGAATAACAAGTTTAGAAACCCCACGGTGGCGCCTACGCATC<br>TCCGGGGGTAGAGCACTGTTTCGGCAAGGGGGTCATCCCGACTTACCAACCCG<br>ATGCAAACTGCGAATACCGGAGAATGTTATCACGGGAGACACACGGCGGGT	AE015928 (4515891-4515973)<br>AB019138 (192-363)<br>AC_000008 (18885-19000)<br>DQ682619 (847-954)	475	EC23S857<br>HAdV<br>Mnif

S2. Fecal sludge sampling apparatus

We built a simple apparatus consisting of a light-weight metal broom handle to which we affixed a ¾ inch PVC “tee” fitting using super glue on one end. A long, sterile, plastic sample bag was secured over the “tee” fitting end with a cable tie to the bottom portion of the shaft as a barrier against the latrine sludge. A sterile 50 mL conical centrifuge tube was attached to the “tee” fitting, outside the protective sampling bag and perpendicular to the sampler shaft, with a pair of cable ties. The apparatus was lowered into the latrine and a scraping motion was used to fill the open centrifuge tube from multiple locations on the sludge surface. After carefully lifting the sampling device out of the pit, the tube screw-cap was replaced and the tube was allowed to drop into a new sterile plastic sample bag by cutting the cable ties attaching the tube to the “tee” fitting with a razor blade. The protective sampling bag was removed from the shaft in a similar manner and the entire device was immediately sanitized with 10% bleach and 70% ethanol.

S3. DNA isolation from fecal samples for MST assay validation

DNA was extracted from animal feces and latrine sludge in Maputo using the FastPrep SPIN Kit for Soils (MP Biomedicals, Santa Ana, CA, USA). After lysing 500 mg thawed fecal sample in the supplied bead tubes by vortexing at maximum speed for 15 minutes, we completed the extractions according to the manufacturer protocol using a final elution volume of 70 µL. Eluted DNA was treated with 17.5 µL DNAstable Plus and maintained at room temperature for up to 14 days during transport to the United States, after which samples were stored at 4 °C and analyzed within 6 months. Latrine samples were extracted in duplicate, and an extraction blank was processed with each sample batch for a total of four negative extraction controls (NEC).

S4. Assay diagnostic performance calculations

For each candidate assay, we counted the true positive (TP) and false negative (FN) fecal samples from its associated animal host, as well as the true negative (TN) and false positive (FP) samples from non-associated fecal sources. We characterized diagnostic performance as the proportion of host samples correctly identified (sensitivity), the proportion of non-host samples in which the microbial target was not detected (specificity), and the proportion of all samples correctly identified (accuracy), as follows:

$$sensitivity = \frac{TP}{TP + FN} \quad \text{(S1)}$$

$$specificity = \frac{TN}{TN + FP} \quad \text{(S2)}$$

$$accurracy = \frac{TP + TN}{TP + FP + TN + FN} \quad \text{(S3)}$$

S5. Processing of environmental samples

Elution of surface swabs was accomplished through vigorous manual shaking of the collection tubes containing swabs and Ringer's solution for 60 seconds (Pickering et al., 2012). We eluted soil samples by adding 1 g wet soil to 100 mL sterile, distilled water in a sterile sample bag and vigorously shaking by hand for 60 seconds. After settling for 15 minutes, the supernatant was used for filtrations (Boehm et al., 2009; Pickering et al., 2012).

To culture *E. coli*, filters were placed on sterile cellulose pads (Pall, Port Washington, NY, USA) saturated with modified mTEC broth (HiMedia, Mumbai, India) in sterile 50 mm

metal plates and incubated at 44.5 ± 0.5 °C for 22 – 26 hours. Approximately 25 mL sterile PBS was added to the filter column before adding any sample volumes of 10 mL or less.

Following membrane filtration for culture-based *E. coli* enumeration, we filtered a larger volume of sample through the same column for molecular analysis. The polycarbonate filters were folded into 2 mL cryovials and immediately archived at -80 °C. Filters were transported frozen on dry ice from Maputo to the United States and stored at -80 °C until DNA extraction, with the exception of eight surface swab filters that experienced room temperature conditions for approximately 24 hours before extraction.

S6. Analysis of unextracted reference material

In addition to the calibration curves constructed using artificial plasmid standards subjected to the DNA extraction procedures, we also prepared three unextracted positive controls with 2×10^6 copies/µL of each artificial plasmid standard and constructed dilution series corresponding to $10^7, 10^5, 10^4, 10^3, 10^2, 5 \times 10^1, 10^1,$ and 5×10^0 copies per 5 µL reaction. For each target, we analyzed each of the three dilution series in triplicate in three separate qPCR instrument runs, for 27 total reactions at each concentration—nine reactions from each dilution series on each of the three plates. To relate these separate instrument runs to the analysis of environmental samples, each extracted PC was re-assayed alongside the unextracted dilution series in duplicate reactions corresponding to 10_5 pre-extraction copies.

S7. Survival modeling to estimate detection limits and extraction efficiency

Theoretical lower limits of detection (tLLoD) were calculated using survival models to estimate the target concentration corresponding to a 95% probability of amplification, a common definition of LLoD for qPCR assays that requires substantial resources to establish empirically (Bustin et al., 2009; Stokdyk et al., 2016). Recognizing that not every copy of the target gene

will successfully amplify but assuming each copy has an independent and identical probability of doing so, we estimated an exponential dose-response relationship between target concentration and detection in the serial dilution series of standard reference material (Verbyla et al., 2016). For each reaction containing d_i $\log_{10}$ copies of the target, the detection status x_i follows a Bernoulli distribution with probability p_i given by

$$p_i = 1 - e^{-d_i r} \tag{S4}$$

where the survival coefficient r is the probability that each copy amplifies (Schmidt et al., 2013). We estimated r and solved for D, the $\log_{10}$ copy number per reaction for which $p_i = 0.95$, using Markov chain Monte Carlo (MCMC) implemented in JAGS with a uniform $\text{Beta}(1,1)$ prior on r and three chains of 2000 warmup and 4000 sampling iterations each (Plummer, 2003). We characterized tLLoD for each assay as the mean and 95% credible interval (CI) of the posterior distribution of D.

We used the unextracted standard dilution series, the dilution series of PCs extracted with the PowerSoil kit, and the dilution series of GeneRite-extracted PCs to estimate three separate tLLoDs for each target. Unextracted tLLoDs correspond to the minimum target concentration in individual reaction wells for reliable detection, while tLLoDs from the extracted dilution series reflect the number of target copies that must be present on the sample filter to ensure amplification following target loss during extraction. We estimated kit-specific extraction efficiency, the proportion of DNA recovered following extraction, as the ratio of tLLoD posterior distributions from unextracted and extracted reactions.

S8. Calibration curve and extraction efficiency estimates

Averaged across all batches and plates, the calibration curves derived from extracted positive controls were relatively linear ($R^2 > 0.95$), although the amplification efficiency was

somewhat poor for some targets, particularly HF183 (Table S3). Reduced linearity and amplification efficiency were both likely related to the use of reference materials that had been subjected to an extraction procedure, which helps account for target loss during processing when quantifying unknown samples but introduces additional variability. Target loss to extraction procedures was substantial, as indicated by the extraction efficiencies implied by the ratio of extracted and unextracted tLLoDs (Table S4). The GeneRite kit consistently recovered a higher proportion of target DNA than the PowerSoil kit, though the recovery estimates for the GeneRite kit were also more variable than for PowerSoil. While the increased variability was likely partially due to the greater number of PCs extracted with the PowerSoil kit, it may also reflect lower consistency for the GeneRite extraction procedure.

Table S3. Mean (95% CI) estimates of calibration curve parameters

target	intercept	slope	efficiency (%)	R^2
EC23S	47.91 (47.25, 48.64)	-3.50 (-3.64, -3.37)	93.11 (88.12, 98.14)	0.98 (0.97, 0.98)
HF183	47.45 (46.41, 48.58)	-3.85 (-4.07, -3.67)	81.83 (76.07, 87.36)	0.98 (0.97, 0.98)
Mnif	48.77 (47.66, 50.25)	-3.47 (-3.79, -3.23)	94.51 (83.57, 104.0)	0.95 (0.93, 0.95)
GFD	44.87 (43.70, 46.11)	-3.63 (-3.88, -3.40)	88.66 (81.06, 96.85)	0.98 (0.97, 0.98)

Table S4. Mean (95% CI) estimates of target DNA recovery by extraction kit

		tLLoD[a]		implied recovery (%)	
**target**	**unextracted**	**PowerSoil[b]**	**GeneRite[c]**	**PowerSoil[b]**	**GeneRite[c]**
EC23S	2.9 (2.7, 3.1)	3.8 (3.6, 4.0)	3.3 (3.1, 3.5)	12 (6, 23)	42 (19, 80)
HF183	2.6 (2.4, 2.7)	3.3 (3.1, 3.5)	3.2 (3.0, 3.4)	21 (11, 35)	25 (12, 44)
Mnif	3.0 (2.8, 3.2)	3.7 (3.5, 3.9)	3.2 (3.0, 3.4)	20 (10, 36)	57 (28, 100)

[a] theoretical lower limit of detection from survival modelling as $\log_{10}$ gc/5 µL reaction
[b] in samples extracted with Qiagen DNeasy PowerSoil kit
[c] in samples extracted with GeneRite DNA-EZ ST01 kit

S9. Variables assessed in risk factor analysis

Household and compound characteristics identified as potential hazards include observed or reported feces, soiled diapers, standing wastewater, domestic animals in the compound yard, previous compound flooding, and disposal of child feces elsewhere than the latrine. We noted amenities including household floor material and onsite access to latrines, source water points, and electricity, as well as physical characteristics of the latrine, if present. We calculated a household wealth index from survey responses using an asset-based scorecard developed for Mozambique, excluding sanitation-related assets (Knee et al., 2018; Schreiner et al., 2013). Other socio-demographic characteristics assessed include caregiver and household head educational attainment, household size and crowding (> three household members per room), and compound population and density. We represented population density as persons per latrine, per waterpoint, and per 100 m^2 of compound area.

We obtained daily records for mean, minimum, and maximum temperature, mean wind speed, cumulative precipitation, and an indicator of whether any precipitation events occurred. In the case of insubstantial precipitation events, it was possible to both observe precipitation and report zero accumulation on the same day. Because meteorological variables were available only as daily summaries and sampling was conducted primarily in the mornings, we associated each environmental sample with meteorological values for the day prior to collection. We also calculated cumulative precipitation and the number of days with rain events in the week (seven days) and month (30 days) preceding sample collection.

Physical characteristics of each sample were observed during collection or determined during initial laboratory processing, in the case of soil moisture. Observed sample characteristics

include source water point location, water storage container attributes, food preparation surface attributes, soil sun exposure, and soil surface wetness.

Due to limited numbers of observations, we collapsed categorical variables with multiple possible responses into dichotomous variables for the risk factor analysis (Table S5). Where applicable, responses were combined such that the response expected to have the strongest relationship with fecal contamination was compared with all others (e.g., child feces disposal in the latrine compared with all other disposal locations). Otherwise, the most frequent response was compared with all others (e.g., plastic water storage containers compared with all other materials) or subcategories were combined into their parent category (e.g., ownership of different animal types represented as ownership of any animals compared with no animals). Precipitation was somewhat infrequent, so we represented precipitation variables as cumulative sums over both the seven and 30 days preceding the sampling event to obtain positive values and investigate different temporal scales. All continuous variables were scaled to improve interpretability of model estimates for meaningful changes the value of the variable. Non-precipitation variables were also mean-centered to aid model convergence and provide effect estimates relative to the typical household, compound, or sampling day.

Table S5. Description of variables assessed as fecal contamination risk factors

variable	type	definition
feces observed	dichotomous	1 if field team observed human feces or used diapers in the compound at the time of the survey<br>0 otherwise
standing wastewater	dichotomous	1 if field team observed standing waste water in the compound at the time of the survey<br>0 otherwise
flood prone	dichotomous	1 if compound head reported the compound tends to flood when it rains<br>0 otherwise
animal ownership	dichotomous	1 if compound head reported any animals lived in the compound<br>0 otherwise
recent diarrhea	dichotomous	1 if caregiver reported diarrhea in past 7 days for any enrolled child in the household (household-level samples) or compound (compound-level samples)<br>0 otherwise
unsafe feces disposal	dichotomous	1 if caregiver reported disposing of feces anywhere besides the latrine for any enrolled child in the household (household-level samples) or compound (compound-level samples)<br>0 otherwise
latrine covered	dichotomous	1 if field team observed a cover over the compound latrine drophole at the time of survey<br>0 otherwise
latrine slab	dichotomous	1 if compound latrine was constructed with a solid slab or concrete/masonry pedestal<br>0 otherwise
superstructure	dichotomous	1 if compound latrine was surrounded by a wall that provided privacy and security<br>0 otherwise
source water available	dichotomous	1 if compound water source was operational and could sampled at time of survey<br>0 otherwise
primary schooling	dichotomous	1 if household head reported completing primary school (household-level samples), or head of any enrolled household in compound (compound-level samples)<br>0 otherwise
wealth index	continuous	asset-based household wealth index (household-level samples) or mean index of all enrolled households in compound (compound-level samples); theoretical range: 0-100<br>mean-centered, scaled by 1/10 (model estimates obtained for a 10-point increase in wealth index)
crowding	dichotomous	1 if household has more than 3 persons per room<br>0 otherwise
persons per 100 m^2	continuous	number of compound residents per 100 m^2 of compound area<br>mean-centered, standard deviation-scaled

variable	type	definition
persons per latrine	continuous	number of compound residents per latrine drophole<br>mean-centered, standard deviation-scaled
yesterday mean temp	continuous	mean daily temperature in °C for the day before sampling<br>mean-centered, standard deviation-scaled
yesterday max temp	continuous	maximum temperature in °C for the day before sampling<br>mean-centered, standard deviation-scaled
past week rain	continuous	cumulative precipitation over the preceding 7 days in cm
past month rain	continuous	cumulative precipitation over the preceding 30 days in cm
rainy days past week	count	number of days on which any rain was recorded during preceding 7 days (theoretical range: 0-7)
rainy days past month	count	number of days on which any rain was recorded during preceding 30 days (theoretical range: 0-30)
plastic container	dichotomous	1 if household water storage container was made of plastic<br>0 otherwise; e.g, metal or concrete construction material
uncovered container	dichotomous	1 if household water storage container mouth was kept uncovered<br>0 otherwise; e.g., a lid was present covering the container mouth
wide mouth container	dichotomous	1 if household water storage container mouth was sufficiently wide to admit hands and other objects<br>0 otherwise; e.g., the mouth was narrow
extracted by dipping	dichotomous	1 if household stored water was obtained for drinking by dipping an object (e.g., cup) into the container<br>0 otherwise; e.g., water was poured out of the container
bowl surface	dichotomous	1 if household food preparation surface provided was a bowl-like object<br>0 otherwise; e.g., table- or board-like surface
plastic surface	dichotomous	1 if household food preparation surface provided was constructed of plastic<br>0 otherwise; e.g., metal or wood surface
reduced sun exposure	dichotomous	1 if soil was not fully exposed to sunlight (i.e., partially or fully shaded)<br>0 otherwise; e.g., full sun exposure
visibly wet	dichotomous	1 if soil surface was visibly wet at time of collection<br>0 otherwise; e.g., soil surface appeared dry by visual inspection

S10. Tabular summaries of model results

Separate univariable models were fit for each combination of risk factor variable, sample type, and microbial target, with the risk factor variable treated as the exposure variable and the microbial target outcome in a given sample type as the response variable. The association between each risk factor and continuous response variables (cEC and EC23S concentrations) was estimated as the expected change in $\log_{10}$ concentration of the microbial target for a one-unit increase in the value of the characteristic. Similarly, associations with binary response variables (detection of HF183 and any human target) were estimated as the odds ratio of detecting the target given a one-unit increase in the value of the characteristic. In addition to the magnitude of the effect estimate, we characterized the strength of each association using the 95% CI of the effect estimate. Variables for which the 95% CI included the corresponding null value (zero for continuous responses and one for binary responses, each signifying no effect) were considered not to be significant risk factors of contamination by the microbial target in the sample type under consideration. Estimated associations between sanitary, sociodemographic, and meteorological characteristics and microbial outcomes for each sample type and microbial target are presented in Table S6 and estimates for sample type-specific characteristics are provided in Table S7.

Table S6. Estimated associations between sanitary, sociodemographic, and meteorological characteristics and microbial indicators of fecal contamination from univariable, multilevel Bayesian regression using censored linear models for *E. coli* concentration responses and logistic models for human target detection responses

			cEC		EC23S		HF183		any human
**characteristic**	**sample type**	**n**	**Δ $\log_{10}$ cfu (95% CI)**	**n**	**Δ $\log_{10}$ gc (95% CI)**	**n**	**OR (95% CI)**	**n**	**OR (95% CI)**
feces observed	latrine soil	53	-0.02 (-0.62, 0.57)	55	0.12 (-0.54, 0.79)	55	1.7 (0.5, 4.4)	55	2.0 (0.5, 5.5)
	household soil	83	0.34 (-0.12, 0.78)	82	0.18 (-0.28, 0.65)	82	6.8 (0.9, 23.0)	82	4.0 (0.6, 16.2)
	stored water	91	0.52 (-0.18, 1.24)	91	0.58 (0.22, 0.95)	91	10.3 (0.3, 34.0)		
	food surface	90	-0.38 (-1.25, 0.46)	89	-0.20 (-0.65, 0.25)				
standing wastewater	latrine soil	53	0.08 (-0.50, 0.66)	55	0.22 (-0.45, 0.88)	55	1.9 (0.5, 5.0)	55	2.7 (0.7, 7.2)
	household soil	83	0.18 (-0.26, 0.65)	82	0.23 (-0.24, 0.72)	82	198.5 (0.8, 31.9)	82	8.9 (1.2, 48.6)
	stored water	91	0.24 (-0.46, 0.98)	91	-0.05 (-0.48, 0.38)	91	1.6 (0.1, 8.1)		
	food surface	90	-0.13 (-1.04, 0.78)	89	0.05 (-0.42, 0.50)				
flood prone	latrine soil	53	-0.17 (-0.72, 0.36)	55	-0.06 (-0.72, 0.60)	55	1.7 (0.5, 4.5)	55	1.1 (0.3, 2.9)
	household soil	83	0.21 (-0.23, 0.65)	82	0.06 (-0.43, 0.53)	82	4.3 (0.6, 14.6)	82	3.9 (0.7, 15.0)
	stored water	91	0.48 (-0.20, 1.15)	91	0.23 (-0.17, 0.63)	91	7.4 (0.4, 37.7)		
	food surface	90	-0.43 (-1.31, 0.46)	89	-0.02 (-0.48, 0.44)				
animal ownership	latrine soil	53	-0.26 (-0.80, 0.30)	55	0.61 (-0.04, 1.24)	55	4.3 (1.2, 11.4)	55	2.3 (0.7, 6.1)
	household soil	83	0.30 (-0.13, 0.73)	82	0.28 (-0.20, 0.76)	82	3.5 (0.5, 13.0)	82	2.7 (0.5, 10.2)
	stored water	91	0.27 (-0.43, 0.93)	91	0.20 (-0.19, 0.60)	91	5.8 (0.4, 25.3)		
	food surface	90	-0.60 (-1.49, 0.29)	89	-0.10 (-0.56, 0.36)				
recent diarrhea	latrine soil	53	0.30 (-0.62, 1.23)	55	1.15 (0.13, 2.20)	55	1.6 (0.1, 6.3)	55	1.3 (0.1, 5.8)
	household soil	81	0.30 (-0.49, 1.09)	80	-0.46 (-1.11, 0.20)	80	0.2 (0.0, 1.5)	80	0.7 (0.0, 2.7)
	stored water	89	-0.40 (-1.47, 0.67)	89	0.10 (-0.53, 0.71)	89	1.6 (0.0, 7.7)		
	food surface	88	0.60 (-0.90, 2.15)	87	0.50 (-0.14, 1.14)				
unsafe feces disposal	latrine soil	53	0.58 (-0.22, 1.36)	55	-0.02 (-0.97, 0.93)	55	2.4 (0.3, 10.0)	55	1.1 (0.1, 4.1)
	household soil	81	-0.05 (-0.57, 0.49)	80	0.01 (-0.46, 0.50)	80	0.5 (0.1, 1.7)	80	1.7 (0.3, 6.1)
	stored water	89	-0.10 (-0.91, 0.66)	89	-0.02 (-0.43, 0.39)	89	1.5 (0.2, 6.1)		
	food surface	88	-0.60 (-1.73, 0.54)	87	0.12 (-0.34, 0.58)				
latrine covered	latrine soil	53	-0.60 (-1.39, 0.16)	55	-0.18 (-1.08, 0.77)	55	1.3 (0.2, 4.6)	55	0.4 (0.0, 1.5)
	household soil	83	-0.07 (-0.69, 0.55)	82	-0.50 (-1.14, 0.13)	82	0.6 (0.1, 2.5)	82	0.4 (0.0, 1.8)
	stored water	91	-0.76 (-1.68, 0.15)	91	-0.49 (-1.00, 0.04)	91	4.8 (0.1, 22.1)		
	food surface	90	-0.02 (-1.27, 1.23)	89	-0.20 (-0.82, 0.46)				
latrine slab	latrine soil	53	-0.35 (-0.90, 0.22)	55	0.06 (-0.60, 0.73)	55	1.6 (0.5, 4.2)	55	0.7 (0.2, 1.7)

characteristic	sample type	n	cEC Δ log_{10} cfu (95% CI)	n	EC23S Δ log_{10} gc (95% CI)	n	HF183 OR (95% CI)	n	any human OR (95% CI)
	household soil	83	-0.14 (-0.60, 0.32)	82	-0.02 (-0.51, 0.46)	82	1.3 (0.2, 3.8)	82	0.4 (0.0, 1.1)
	stored water	91	0.25 (-0.49, 0.94)	91	-0.08 (-0.50, 0.33)	91	2.0 (0.1, 8.7)		
	food surface	90	-0.58 (-1.51, 0.31)	89	0.48 (0.05, 0.92)				
superstructure	latrine soil	53	-0.35 (-0.94, 0.26)	55	-0.05 (-0.77, 0.65)	55	2.1 (0.5, 5.8)	55	2.0 (0.4, 6.3)
	household soil	83	-0.09 (-0.58, 0.38)	82	-0.01 (-0.56, 0.53)	82	3.8 (0.5, 15.1)	82	3.9 (0.5, 16.3)
	stored water	91	0.06 (-0.71, 0.81)	91	-0.22 (-0.68, 0.22)	91	1.2 (0.0, 5.5)		
	food surface	90	-0.16 (-1.14, 0.82)	89	-0.18 (-0.71, 0.34)				
source water available	latrine soil	53	-0.34 (-0.92, 0.25)	55	0.96 (0.28, 1.66)	55	4.5 (0.8, 15.6)	55	1.6 (0.4, 4.5)
	household soil	83	-0.29 (-0.80, 0.22)	82	-0.17 (-0.71, 0.40)	82	3.6 (0.4, 15.0)	82	1.1 (0.1, 3.9)
	stored water	91	-0.71 (-1.53, 0.10)	91	-0.43 (-0.89, 0.03)	91	4.1 (0.2, 16.1)		
	food surface	90	-0.68 (-1.70, 0.40)	89	-0.05 (-0.58, 0.47)				
primary schooling	latrine soil	53	-0.55 (-1.15, 0.05)	55	0.12 (-0.60, 0.85)	55	1.1 (0.3, 3.1)	55	1.6 (0.4, 4.3)
	household soil	82	-0.13 (-0.56, 0.30)	81	-0.12 (-0.51, 0.26)	81	0.7 (0.1, 2.0)	81	0.2 (0.0, 0.6)
	stored water	90	-0.11 (-0.78, 0.59)	90	-0.06 (-0.46, 0.33)				
	food surface	89	-0.55 (-1.48, 0.35)	88	-0.21 (-0.64, 0.17)				
wealth index	latrine soil	53	-0.14 (-0.48, 0.20)	55	-0.09 (-0.49, 0.31)	55	0.8 (0.4, 1.5)	55	0.7 (0.3, 1.3)
	household soil	82	-0.03 (-0.20, 0.15)	81	-0.10 (-0.26, 0.06)	81	7.4 (1.6, 32.7)	81	1.8 (0.8, 4.8)
	stored water	90	-0.10 (-0.41, 0.19)	90	0.00 (-0.17, 0.16)	90	1.0 (0.4, 2.0)		
	food surface	89	0.10 (-0.31, 0.52)	88	-0.01 (-0.19, 0.16)				
crowding	latrine soil	53	0.12 (-1.00, 1.21)	55	-0.09 (-1.48, 1.26)				
	household soil	82	0.28 (-0.28, 0.87)	81	0.22 (-0.29, 0.78)	81	0.9 (0.1, 3.5)	81	0.9 (0.1, 3.6)
	stored water	90	0.12 (-0.80, 1.05)	90	0.01 (-0.49, 0.51)	90	8.1 (0.4, 45.8)		
	food surface	89	-0.51 (-1.80, 0.74)	88	0.05 (-0.49, 0.60)				
persons per 100 m^2	latrine soil	48	0.17 (-0.14, 0.48)	50	0.35 (0.01, 0.69)	50	1.3 (0.7, 2.2)	50	1.5 (0.8, 2.7)
	household soil	72	0.12 (-0.14, 0.38)	71	0.11 (-0.18, 0.39)	71	0.9 (0.1, 2.5)	71	0.7 (0.1, 1.9)
	stored water	80	-0.28 (-0.66, 0.06)	80	-0.02 (-0.24, 0.21)	80	1.8 (0.5, 6.3)		
	food surface	79	-0.08 (-0.59, 0.43)	79	0.15 (-0.09, 0.39)				
persons per latrine	latrine soil	53	0.10 (-0.17, 0.38)	55	0.41 (0.10, 0.72)	55	1.9 (1.0, 3.5)	55	2.6 (1.1, 5.5)
	household soil	83	0.27 (0.09, 0.46)	82	0.20 (-0.02, 0.42)	82	0.9 (0.3, 1.7)	82	1.7 (0.8, 3.8)
	stored water	91	0.00 (-0.31, 0.31)	91	0.04 (-0.15, 0.21)	91	1.8 (0.5, 5.8)		
	food surface	90	-0.16 (-0.57, 0.23)	89	-0.10 (-0.32, 0.11)				
yesterday mean temp (°C)	latrine soil	54	-0.21 (-0.46, 0.05)	56	-0.02 (-0.33, 0.29)	56	0.6 (0.3, 1.1)	56	0.9 (0.5, 1.6)
	household soil	85	-0.04 (-0.27, 0.19)	84	0.04 (-0.19, 0.26)	84	0.6 (0.2, 1.1)	84	0.5 (0.1, 1.1)

characteristic	sample type	n	cEC<br>Δ $\log_{10}$ cfu (95% CI)	n	EC23S<br>Δ $\log_{10}$ gc (95% CI)	n	HF183<br>OR (95% CI)	n	any human<br>OR (95% CI)
	stored water	91	-0.08 (-0.44, 0.26)	91	-0.08 (-0.28, 0.12)	91	1.0 (0.2, 2.8)		
	food surface	90	-0.09 (-0.56, 0.38)	89	-0.20 (-0.41, 0.02)				
yesterday max temp (°C)	latrine soil	54	-0.22 (-0.50, 0.06)	56	0.11 (-0.21, 0.43)	56	0.5 (0.2, 0.8)	56	0.8 (0.4, 1.4)
	household soil	85	-0.18 (-0.40, 0.03)	84	-0.11 (-0.34, 0.12)	84	0.8 (0.3, 1.5)	84	0.3 (0.1, 0.7)
	stored water	91	-0.36 (-0.68, -0.03)	91	-0.06 (-0.27, 0.16)	91	1.1 (0.2, 3.1)		
	food surface	90	-0.07 (-0.53, 0.39)	89	0.13 (-0.10, 0.35)				
past week rain (cm)	latrine soil	54	0.00 (-0.11, 0.11)	56	0.03 (-0.10, 0.17)	56	0.9 (0.6, 1.1)	56	1.0 (0.8, 1.3)
	household soil	85	-0.05 (-0.14, 0.04)	84	0.01 (-0.08, 0.10)	84	1.1 (0.7, 1.4)	84	1.0 (0.6, 1.4)
	stored water	91	-0.19 (-0.33, -0.05)	91	0.00 (-0.08, 0.09)	91	0.7 (0.2, 1.3)		
	food surface	90	-0.01 (-0.19, 0.18)	89	0.08 (-0.01, 0.18)				
past month rain (cm)	latrine soil	54	0.07 (-0.01, 0.15)	56	-0.09 (-0.19, 0.00)	56	1.0 (0.8, 1.1)	56	1.1 (0.9, 1.3)
	household soil	85	0.01 (-0.05, 0.08)	84	0.04 (-0.03, 0.11)	84	1.0 (0.8, 1.3)	84	1.2 (0.9, 1.7)
	stored water	91	0.04 (-0.07, 0.15)	91	0.00 (-0.06, 0.06)	91	0.7 (0.3, 1.1)		
	food surface	90	-0.01 (-0.16, 0.12)	89	0.02 (-0.05, 0.09)				
rainy days past week	latrine soil	54	0.19 (-0.10, 0.48)	56	0.05 (-0.33, 0.44)	56	1.4 (0.7, 2.5)	56	2.9 (1.2, 6.3)
	household soil	85	0.26 (0.02, 0.50)	84	0.21 (-0.03, 0.47)	84	0.9 (0.3, 2.0)	84	1.5 (0.5, 3.9)
	stored water	91	0.43 (0.05, 0.81)	91	0.02 (-0.22, 0.24)	91	1.9 (0.5, 6.0)		
	food surface	90	0.43 (-0.05, 0.92)	89	-0.14 (-0.40, 0.11)				
rainy days past month	latrine soil	54	0.02 (-0.12, 0.16)	56	0.00 (-0.17, 0.16)	56	1.2 (0.9, 1.6)	56	1.4 (1.1, 2.0)
	household soil	85	0.12 (0.02, 0.23)	84	0.13 (0.03, 0.24)	84	1.1 (0.7, 1.7)	84	1.8 (1.2, 2.9)
	stored water	91	0.26 (0.08, 0.44)	91	0.01 (-0.09, 0.12)	91	1.4 (0.7, 3.2)		
	food surface	90	0.15 (-0.08, 0.39)	89	-0.23 (-0.32, -0.13)				

Table S7. Estimated associations between environmental sample characteristics and microbial indicators of fecal contamination from univariable, multilevel Bayesian regression using censored linear models for *E. coli* concentration responses and logistic models for human target detection responses

			cEC		EC23S		HF183		any human
**sample type**	**characteristic**	**n**	**Δ log_{10} cfu (95% CI)**	**n**	**Δ log_{10} gc (95% CI)**	**n**	**OR (95% CI)**	**n**	**OR (95% CI)**
latrine soil	reduced sun exposure	49	0.23 (-0.43, 0.91)	49	0.29 (-0.48, 1.05)	49	3.52 (0.70, 11.71)	49	1.67 (0.33, 4.99)
latrine soil	visibly wet	49	0.67 (0.09, 1.26)	49	1.08 (0.45, 1.74)	49	38.53 (2.98, 238.23)	49	6.61 (1.54, 20.18)
household soil	reduced sun exposure	85	0.20 (-0.31, 0.72)	84	0.04 (-0.46, 0.54)	84	0.59 (0.16, 1.66)	84	0.75 (0.21, 1.99)
household soil	visibly wet	83	0.45 (0.07, 0.85)	82	0.27 (-0.13, 0.68)	82	1.08 (0.36, 2.61)	82	1.17 (0.46, 2.53)
stored water	extracted by dipping	89	0.37 (-0.29, 1.05)	89	-0.18 (-0.59, 0.21)	89	5.27 (0.88, 19.37)	88	4.85 (0.81, 19.69)
stored water	plastic container	90	-0.50 (-1.75, 0.69)	90	-0.38 (-1.09, 0.35)	90	0.31 (0.04, 1.20)	89	0.30 (0.03, 1.14)
stored water	uncovered container	89	-0.10 (-0.77, 0.57)	89	0.26 (-0.11, 0.64)	89	2.63 (0.74, 6.71)	88	2.81 (0.69, 7.44)
stored water	wide mouth container	89	0.38 (-0.32, 1.06)	89	-0.19 (-0.58, 0.20)	89	4.72 (0.82, 18.95)	88	4.27 (0.75, 15.53)
food surface	bowl surface	90	0.05 (-1.59, 1.56)	89	-1.11 (-1.76, -0.47)				
food surface	plastic surface	90	0.97 (-0.32, 2.22)	89	-0.82 (-1.37, -0.25)				

S11. Supplementary References

Agarwala, R., Barrett, T., Beck, J., Benson, D.A., Bollin, C., Bolton, E., Bourexis, D., Brister, J.R., Bryant, S.H., Canese, K., Charowhas, C., Clark, K., Dicuccio, M., Dondoshansky, I., Federhen, S., Feolo, M., Funk, K., Geer, L.Y., Gorelenkov, V., Hoeppner, M., Holmes, B., Johnson, M., Khotomlianski, V., Kimchi, A., Kimelman, M., Kitts, P., Klimke, W., Krasnov, S., Kuznetsov, A., Landrum, M.J., Landsman, D., Lee, J.M., Lipman, D.J., Lu, Z., Madden, T.L., Madej, T., Marchler-Bauer, A., Karsch-Mizrachi, I., Murphy, T., Orris, R., Ostell, J., O'sullivan, C., Panchenko, A., Phan, L., Preuss, D., Pruitt, K.D., Rodarmer, K., Rubinstein, W., Sayers, E., Schneider, V., Schuler, G.D., Sherry, S.T., Sirotkin, K., Siyan, K., Slotta, D., Soboleva, A., Soussov, V., Starchenko, G., Tatusova, T.A., Todorov, K., Trawick, B.W., Vakatov, D., Wang, Y., Ward, M., Wilbur, W.J., Yaschenko, E., Zbicz, K., 2016. Database resources of the National Center for Biotechnology Information. Nucleic Acids Res. 44, D7–D19. doi:10.1093/nar/gkv1290

Boehm, A.B., Griffith, J., McGee, C., Edge, T.A., Solo-Gabriele, H.M., Whitman, R., Cao, Y., Getrich, M., Jay, J.A., Ferguson, D., Goodwin, K.D., Lee, C.M., Madison, M., Weisberg, S.B., 2009. Faecal indicator bacteria enumeration in beach sand: a comparison study of extraction methods in medium to coarse sands. J. Appl. Microbiol. 107, 1740–1750. doi:10.1111/j.1365-2672.2009.04440.x

Bustin, S.A., Benes, V., Garson, J.A., Hellemans, J., Huggett, J., Kubista, M., Mueller, R., Nolan, T., Pfaffl, M.W., Shipley, G.L., Vandesompele, J., Wittwer, C.T., 2009. The MIQE Guidelines: Minimum Information for Publication of Quantitative Real-Time PCR Experiments. Clin. Chem. 55, 611–622. doi:10.1373/clinchem.2008.112797

Chern, E.C., Siefring, S., Paar, J., Doolittle, M., Haugland, R.A., 2011. Comparison of quantitative PCR assays for Escherichia coli targeting ribosomal RNA and single copy genes. Lett. Appl. Microbiol. 52, 298–306. doi:10.1111/j.1472-765X.2010.03001.x

Clark, K., Karsch-Mizrachi, I., Lipman, D.J., Ostell, J., Sayers, E.W., 2016. GenBank. Nucleic Acids Res. 44, D67–D72. doi:10.1093/nar/gkv1276

Green, H.C., Dick, L.K., Gilpin, B., Samadpour, M., Field, K.G., 2012. Genetic markers for rapid PCR-based identification of gull, Canada goose, duck, and chicken fecal contamination in water. Appl. Environ. Microbiol. 78, 503–10. doi:10.1128/AEM.05734-11

Green, H.C., Haugland, R.A., Varma, M., Millen, H.T., Borchardt, M.A., Field, K.G., Walters, W.A., Knight, R., Sivaganesan, M., Kelty, C.A., Shanks, O.C., 2014. Improved HF183 Quantitative Real-Time PCR Assay for Characterization of Human Fecal Pollution in Ambient Surface Water Samples. Appl. Environ. Microbiol. 80, 3086–3094. doi:10.1128/AEM.04137-13

Haugland, R. a, Siefring, S.C., Wymer, L.J., Brenner, K.P., Dufour, A.P., 2005. Comparison of Enterococcus measurements in freshwater at two recreational beaches by quantitative polymerase chain reaction and membrane filter culture analysis. Water Res. 39, 559–568. doi:10.1016/j.watres.2004.11.011

Haugland, R. a, Varma, M., Sivaganesan, M., Kelty, C., Peed, L., Shanks, O.C., 2010. Evaluation of genetic markers from the 16S rRNA gene V2 region for use in quantitative detection of selected Bacteroidales species and human fecal waste by qPCR. Syst. Appl. Microbiol. 33, 348–357. doi:10.1016/j.syapm.2010.06.001

Johnston, C., Ufnar, J. a, Griffith, J.F., Gooch, J. a, Stewart, J.R., 2010. A real-time qPCR assay for the detection of the nifH gene of Methanobrevibacter smithii, a potential indicator of

sewage pollution. J. Appl. Microbiol. 109, 1946–56. doi:10.1111/j.1365-2672.2010.04824.x

Jothikumar, N., Cromeans, T.L., Vincent, R., Lu, X., Sobsey, M.D., Erdman, D.D., Hill, V.R., 2005. Quantitative Real-Time PCR Assays for Detection of Human Adenoviruses and Identification of Serotypes 40 and 41 Quantitative Real-Time PCR Assays for Detection of Human Adenoviruses and Identification of Serotypes 40 and 41. Appl Env. Microbiol. 6, 3131–6. doi:10.1128/AEM.71.6.3131

Kildare, B.J., Leutenegger, C.M., McSwain, B.S., Bambic, D.G., Rajal, V.B., Wuertz, S., 2007. 16S rRNA-based assays for quantitative detection of universal, human-, cow-, and dog-specific fecal Bacteroidales: A Bayesian approach. Water Res. 41, 3701–3715. doi:10.1016/j.watres.2007.06.037

Knee, J., Sumner, T., Adriano, Z., Berendes, D., de Bruijn, E., Schmidt, W., Nalá, R., Cumming, O., Brown, J., 2018. Risk factors for childhood enteric infection in urban Maputo, Mozambique: A cross-sectional study. PLoS Negl. Trop. Dis. 12, e0006956. doi:10.1371/journal.pntd.0006956

Kodani, M., Winchell, J.M., 2012. Engineered combined-positive-control template for real-time reverse transcription-PCR in multiple-pathogen-detection assays. J. Clin. Microbiol. 50, 1057–1060. doi:10.1128/JCM.05987-11

Liu, J., Gratz, J., Amour, C., Kibiki, G., Becker, S., Janaki, L., Verweij, J.J., Taniuchi, M., Sobuz, S.U., Haque, R., Haverstick, D.M., Houpt, E.R., 2013. A Laboratory-Developed TaqMan Array Card for Simultaneous Detection of 19 Enteropathogens. J. Clin. Microbiol. 51, 472–480. doi:10.1128/JCM.02658-12

Pickering, A.J., Julian, T.R., Marks, S.J., Mattioli, M.C., Boehm, A.B., Schwab, K.J., Davis, J., 2012. Fecal Contamination and Diarrheal Pathogens on Surfaces and in Soils among Tanzanian Households with and without Improved Sanitation. Environ. Sci. Technol. 46, 5736–5743. doi:10.1021/es300022c

Plummer, M., 2003. JAGS: A program for analysis of Bayesian graphical models using Gibbs sampling.

Schmidt, P.J., Pintar, K.D.M., Fazil, A.M., Topp, E., 2013. Harnessing the Theoretical Foundations of the Exponential and Beta-Poisson Dose-Response Models to Quantify Parameter Uncertainty Using Markov Chain Monte Carlo. Risk Anal. 33, 1677–1693. doi:10.1111/risa.12006

Schreiner, M., Nsthandoca, H., Lory, D., Dd, D., Yy, M.M., 2013. A Simple Poverty Scorecard for Mozambique.

Stokdyk, J.P., Firnstahl, A.D., Spencer, S.K., Burch, T.R., Borchardt, M.A., 2016. Determining the 95% limit of detection for waterborne pathogen analyses from primary concentration to qPCR. Water Res. 96, 105–113. doi:10.1016/j.watres.2016.03.026

Verbyla, M.E., Symonds, E.M., Kafle, R.C., Cairns, M.R., Iriarte, M., Mercado Guzmán, A., Coronado, O., Breitbart, M., Ledo, C., Mihelcic, J.R., 2016. Managing Microbial Risks from Indirect Wastewater Reuse for Irrigation in Urbanizing Watersheds. Environ. Sci. Technol. 50, 6803–6813. doi:10.1021/acs.est.5b05398

Weidhaas, J.L., Macbeth, T.W., Olsen, R.L., Sadowsky, M.J., Norat, D., Harwood, V.J., 2010. Identification of a Brevibacterium marker gene specific to poultry litter and development of a quantitative PCR assay. J Appl Microbiol 109, 334–347. doi:10.1111/j.1365-2672.2010.04666.x
