# Supplementary material for: Human fecal contamination of water, soil, and surfaces in households sharing poor-quality sanitation facilities in Maputo, Mozambique
Source: Int J Hyg Environ Health. Author manuscript; Available in PMC 2020 May 1. (PMC7174141; doi:10.1016/j.ijheh.2020.113496)
Supplement: Supplementary file 1 [file NIHMS1570378-supplement-Table_S3.docx]

Table S3. Mean (95% CI) estimates of calibration curve parameters

| **target** | **intercept** | **slope** | **efficiency (%)** | **R**^**2**^ |
| --- | --- | --- | --- | --- |
| EC23S | 47.91 (47.25, 48.64) | -3.50 (-3.64, -3.37) | 93.11 (88.12, 98.14) | 0.98 (0.97, 0.98) |
| HF183 | 47.45 (46.41, 48.58) | -3.85 (-4.07, -3.67) | 81.83 (76.07, 87.36) | 0.98 (0.97, 0.98) |
| Mnif | 48.77 (47.66, 50.25) | -3.47 (-3.79, -3.23) | 94.51 (83.57, 104.0) | 0.95 (0.93, 0.95) |
| GFD | 44.87 (43.70, 46.11) | -3.63 (-3.88, -3.40) | 88.66 (81.06, 96.85) | 0.98 (0.97, 0.98) |
