# Supplementary material for: Human fecal contamination of water, soil, and surfaces in households sharing poor-quality sanitation facilities in Maputo, Mozambique
Source: Int J Hyg Environ Health. Author manuscript; Available in PMC 2020 May 1. (PMC7174141; doi:10.1016/j.ijheh.2020.113496)
Supplement: Supplementary file 2 [file NIHMS1570378-supplement-Table_S5.docx]

Table S5. Description of variables assessed as fecal contamination risk factors

| **variable** | **type** | **definition** |
| --- | --- | --- |
| feces observed | dichotomous | 1 if field team observed human feces or used diapers in the compound at the time of the survey 0 otherwise |
| standing wastewater | dichotomous | 1 if field team observed standing waste water in the compound at the time of the survey 0 otherwise |
| flood prone | dichotomous | 1 if compound head reported the compound tends to flood when it rains 0 otherwise |
| animal ownership | dichotomous | 1 if compound head reported any animals lived in the compound 0 otherwise |
| recent diarrhea | dichotomous | 1 if caregiver reported diarrhea in past 7 days for any enrolled child in the household (household-level samples) or compound (compound-level samples) 0 otherwise |
| unsafe feces disposal | dichotomous | 1 if caregiver reported disposing of feces anywhere besides the latrine for any enrolled child in the household (household-level samples) or compound (compound-level samples) 0 otherwise |
| latrine covered | dichotomous | 1 if field team observed a cover over the compound latrine drophole at the time of survey 0 otherwise |
| latrine slab | dichotomous | 1 if compound latrine was constructed with a solid slab or concrete/masonry pedestal 0 otherwise |
| superstructure | dichotomous | 1 if compound latrine was surrounded by a wall that provided privacy and security 0 otherwise |
| source water available | dichotomous | 1 if compound water source was operational and could sampled at time of survey 0 otherwise |
| primary schooling | dichotomous | 1 if household head reported completing primary school (household-level samples), or head of any enrolled household in compound (compound-level samples) 0 otherwise |
| wealth index | continuous | asset-based household wealth index (household-level samples) or mean index of all enrolled households in compound (compound-level samples); theoretical range: 0-100 mean-centered, scaled by 1/10 (model estimates obtained for a 10-point increase in wealth index) |
| crowding | dichotomous | 1 if household has more than 3 persons per room 0 otherwise |
| persons per 100 m^2^ | continuous | number of compound residents per 100 m^2^ of compound area mean-centered, standard deviation-scaled |
| persons per latrine | continuous | number of compound residents per latrine drophole mean-centered, standard deviation-scaled |
| yesterday mean temp | continuous | mean daily temperature in °C for the day before sampling mean-centered, standard deviation-scaled |
| yesterday max temp | continuous | maximum temperature in °C for the day before sampling mean-centered, standard deviation-scaled |
| past week rain | continuous | cumulative precipitation over the preceding 7 days in cm |
| past month rain | continuous | cumulative precipitation over the preceding 30 days in cm |
| rainy days past week | count | number of days on which any rain was recorded during preceding 7 days (theoretical range: 0-7) |
| rainy days past month | count | number of days on which any rain was recorded during preceding 30 days (theoretical range: 0-30) |
| plastic container | dichotomous | 1 if household water storage container was made of plastic 0 otherwise; e.g, metal or concrete construction material |
| uncovered container | dichotomous | 1 if household water storage container mouth was kept uncovered 0 otherwise; e.g., a lid was present covering the container mouth |
| wide mouth container | dichotomous | 1 if household water storage container mouth was sufficiently wide to admit hands and other objects 0 otherwise; e.g., the mouth was narrow |
| extracted by dipping | dichotomous | 1 if household stored water was obtained for drinking by dipping an object (e.g., cup) into the container 0 otherwise; e.g., water was poured out of the container |
| bowl surface | dichotomous | 1 if household food preparation surface provided was a bowl-like object 0 otherwise; e.g., table- or board-like surface |
| plastic surface | dichotomous | 1 if household food preparation surface provided was constructed of plastic 0 otherwise; e.g., metal or wood surface |
| reduced sun exposure | dichotomous | 1 if soil was not fully exposed to sunlight (i.e., partially or fully shaded) 0 otherwise; e.g., full sun exposure |
| visibly wet | dichotomous | 1 if soil surface was visibly wet at time of collection 0 otherwise; e.g., soil surface appeared dry by visual inspection |
