# Supplementary material for: Human fecal contamination of water, soil, and surfaces in households sharing poor-quality sanitation facilities in Maputo, Mozambique
Source: Int J Hyg Environ Health. Author manuscript; Available in PMC 2020 May 1. (PMC7174141; doi:10.1016/j.ijheh.2020.113496)
Supplement: Supplementary file 3 [file NIHMS1570378-supplement-Table_S6.docx]

Table S6. Estimated associations between sanitary, sociodemographic, and meteorological characteristics and microbial indicators of fecal contamination from univariable, multilevel Bayesian regression using censored linear models for *E. coli* concentration responses and logistic models for human target detection responses

|  |  | **cEC** |  | **EC23S** |  | **HF183** |  | **any human** |  |
| --- | --- | --- | --- | --- | --- | --- | --- | --- | --- |
| **characteristic** | **sample type** | **n** | **Δ log**_**10**_ **cfu (95% CI)** | **n** | **Δ log**_**10**_ **gc (95% CI)** | **n** | **OR (95% CI)** | **n** | **OR (95% CI)** |
| feces observed | latrine soil | 53 | -0.02 (-0.62, 0.57) | 55 | 0.12 (-0.54, 0.79) | 55 | 1.7 (0.5, 4.4) | 55 | 2.0 (0.5, 5.5) |
|  | household soil | 83 | 0.34 (-0.12, 0.78) | 82 | 0.18 (-0.28, 0.65) | 82 | 6.8 (0.9, 23.0) | 82 | 4.0 (0.6, 16.2) |
|  | stored water | 91 | 0.52 (-0.18, 1.24) | 91 | 0.58 (0.22, 0.95) | 91 | 10.3 (0.3, 34.0) |  |  |
|  | food surface | 90 | -0.38 (-1.25, 0.46) | 89 | -0.20 (-0.65, 0.25) |  |  |  |  |
| standing wastewater | latrine soil | 53 | 0.08 (-0.50, 0.66) | 55 | 0.22 (-0.45, 0.88) | 55 | 1.9 (0.5, 5.0) | 55 | 2.7 (0.7, 7.2) |
|  | household soil | 83 | 0.18 (-0.26, 0.65) | 82 | 0.23 (-0.24, 0.72) | 82 | 198.5 (0.8, 31.9) | 82 | 8.9 (1.2, 48.6) |
|  | stored water | 91 | 0.24 (-0.46, 0.98) | 91 | -0.05 (-0.48, 0.38) | 91 | 1.6 (0.1, 8.1) |  |  |
|  | food surface | 90 | -0.13 (-1.04, 0.78) | 89 | 0.05 (-0.42, 0.50) |  |  |  |  |
| flood prone | latrine soil | 53 | -0.17 (-0.72, 0.36) | 55 | -0.06 (-0.72, 0.60) | 55 | 1.7 (0.5, 4.5) | 55 | 1.1 (0.3, 2.9) |
|  | household soil | 83 | 0.21 (-0.23, 0.65) | 82 | 0.06 (-0.43, 0.53) | 82 | 4.3 (0.6, 14.6) | 82 | 3.9 (0.7, 15.0) |
|  | stored water | 91 | 0.48 (-0.20, 1.15) | 91 | 0.23 (-0.17, 0.63) | 91 | 7.4 (0.4, 37.7) |  |  |
|  | food surface | 90 | -0.43 (-1.31, 0.46) | 89 | -0.02 (-0.48, 0.44) |  |  |  |  |
| animal ownership | latrine soil | 53 | -0.26 (-0.80, 0.30) | 55 | 0.61 (-0.04, 1.24) | 55 | 4.3 (1.2, 11.4) | 55 | 2.3 (0.7, 6.1) |
|  | household soil | 83 | 0.30 (-0.13, 0.73) | 82 | 0.28 (-0.20, 0.76) | 82 | 3.5 (0.5, 13.0) | 82 | 2.7 (0.5, 10.2) |
|  | stored water | 91 | 0.27 (-0.43, 0.93) | 91 | 0.20 (-0.19, 0.60) | 91 | 5.8 (0.4, 25.3) |  |  |
|  | food surface | 90 | -0.60 (-1.49, 0.29) | 89 | -0.10 (-0.56, 0.36) |  |  |  |  |
| recent diarrhea | latrine soil | 53 | 0.30 (-0.62, 1.23) | 55 | 1.15 (0.13, 2.20) | 55 | 1.6 (0.1, 6.3) | 55 | 1.3 (0.1, 5.8) |
|  | household soil | 81 | 0.30 (-0.49, 1.09) | 80 | -0.46 (-1.11, 0.20) | 80 | 0.2 (0.0, 1.5) | 80 | 0.7 (0.0, 2.7) |
|  | stored water | 89 | -0.40 (-1.47, 0.67) | 89 | 0.10 (-0.53, 0.71) | 89 | 1.6 (0.0, 7.7) |  |  |
|  | food surface | 88 | 0.60 (-0.90, 2.15) | 87 | 0.50 (-0.14, 1.14) |  |  |  |  |
| unsafe feces disposal | latrine soil | 53 | 0.58 (-0.22, 1.36) | 55 | -0.02 (-0.97, 0.93) | 55 | 2.4 (0.3, 10.0) | 55 | 1.1 (0.1, 4.1) |
|  | household soil | 81 | -0.05 (-0.57, 0.49) | 80 | 0.01 (-0.46, 0.50) | 80 | 0.5 (0.1, 1.7) | 80 | 1.7 (0.3, 6.1) |
|  | stored water | 89 | -0.10 (-0.91, 0.66) | 89 | -0.02 (-0.43, 0.39) | 89 | 1.5 (0.2, 6.1) |  |  |
|  | food surface | 88 | -0.60 (-1.73, 0.54) | 87 | 0.12 (-0.34, 0.58) |  |  |  |  |
| latrine covered | latrine soil | 53 | -0.60 (-1.39, 0.16) | 55 | -0.18 (-1.08, 0.77) | 55 | 1.3 (0.2, 4.6) | 55 | 0.4 (0.0, 1.5) |
|  | household soil | 83 | -0.07 (-0.69, 0.55) | 82 | -0.50 (-1.14, 0.13) | 82 | 0.6 (0.1, 2.5) | 82 | 0.4 (0.0, 1.8) |
|  | stored water | 91 | -0.76 (-1.68, 0.15) | 91 | -0.49 (-1.00, 0.04) | 91 | 4.8 (0.1, 22.1) |  |  |
|  | food surface | 90 | -0.02 (-1.27, 1.23) | 89 | -0.20 (-0.82, 0.46) |  |  |  |  |
| latrine slab | latrine soil | 53 | -0.35 (-0.90, 0.22) | 55 | 0.06 (-0.60, 0.73) | 55 | 1.6 (0.5, 4.2) | 55 | 0.7 (0.2, 1.7) |
|  | household soil | 83 | -0.14 (-0.60, 0.32) | 82 | -0.02 (-0.51, 0.46) | 82 | 1.3 (0.2, 3.8) | 82 | 0.4 (0.0, 1.1) |
|  | stored water | 91 | 0.25 (-0.49, 0.94) | 91 | -0.08 (-0.50, 0.33) | 91 | 2.0 (0.1, 8.7) |  |  |
|  | food surface | 90 | -0.58 (-1.51, 0.31) | 89 | 0.48 (0.05, 0.92) |  |  |  |  |
| superstructure | latrine soil | 53 | -0.35 (-0.94, 0.26) | 55 | -0.05 (-0.77, 0.65) | 55 | 2.1 (0.5, 5.8) | 55 | 2.0 (0.4, 6.3) |
|  | household soil | 83 | -0.09 (-0.58, 0.38) | 82 | -0.01 (-0.56, 0.53) | 82 | 3.8 (0.5, 15.1) | 82 | 3.9 (0.5, 16.3) |
|  | stored water | 91 | 0.06 (-0.71, 0.81) | 91 | -0.22 (-0.68, 0.22) | 91 | 1.2 (0.0, 5.5) |  |  |
|  | food surface | 90 | -0.16 (-1.14, 0.82) | 89 | -0.18 (-0.71, 0.34) |  |  |  |  |
| source water available | latrine soil | 53 | -0.34 (-0.92, 0.25) | 55 | 0.96 (0.28, 1.66) | 55 | 4.5 (0.8, 15.6) | 55 | 1.6 (0.4, 4.5) |
|  | household soil | 83 | -0.29 (-0.80, 0.22) | 82 | -0.17 (-0.71, 0.40) | 82 | 3.6 (0.4, 15.0) | 82 | 1.1 (0.1, 3.9) |
|  | stored water | 91 | -0.71 (-1.53, 0.10) | 91 | -0.43 (-0.89, 0.03) | 91 | 4.1 (0.2, 16.1) |  |  |
|  | food surface | 90 | -0.68 (-1.70, 0.40) | 89 | -0.05 (-0.58, 0.47) |  |  |  |  |
| primary schooling | latrine soil | 53 | -0.55 (-1.15, 0.05) | 55 | 0.12 (-0.60, 0.85) | 55 | 1.1 (0.3, 3.1) | 55 | 1.6 (0.4, 4.3) |
|  | household soil | 82 | -0.13 (-0.56, 0.30) | 81 | -0.12 (-0.51, 0.26) | 81 | 0.7 (0.1, 2.0) | 81 | 0.2 (0.0, 0.6) |
|  | stored water | 90 | -0.11 (-0.78, 0.59) | 90 | -0.06 (-0.46, 0.33) |  |  |  |  |
|  | food surface | 89 | -0.55 (-1.48, 0.35) | 88 | -0.21 (-0.64, 0.17) |  |  |  |  |
| wealth index | latrine soil | 53 | -0.14 (-0.48, 0.20) | 55 | -0.09 (-0.49, 0.31) | 55 | 0.8 (0.4, 1.5) | 55 | 0.7 (0.3, 1.3) |
|  | household soil | 82 | -0.03 (-0.20, 0.15) | 81 | -0.10 (-0.26, 0.06) | 81 | 7.4 (1.6, 32.7) | 81 | 1.8 (0.8, 4.8) |
|  | stored water | 90 | -0.10 (-0.41, 0.19) | 90 | 0.00 (-0.17, 0.16) | 90 | 1.0 (0.4, 2.0) |  |  |
|  | food surface | 89 | 0.10 (-0.31, 0.52) | 88 | -0.01 (-0.19, 0.16) |  |  |  |  |
| crowding | latrine soil | 53 | 0.12 (-1.00, 1.21) | 55 | -0.09 (-1.48, 1.26) |  |  |  |  |
|  | household soil | 82 | 0.28 (-0.28, 0.87) | 81 | 0.22 (-0.29, 0.78) | 81 | 0.9 (0.1, 3.5) | 81 | 0.9 (0.1, 3.6) |
|  | stored water | 90 | 0.12 (-0.80, 1.05) | 90 | 0.01 (-0.49, 0.51) | 90 | 8.1 (0.4, 45.8) |  |  |
|  | food surface | 89 | -0.51 (-1.80, 0.74) | 88 | 0.05 (-0.49, 0.60) |  |  |  |  |
| persons per 100 m^2^ | latrine soil | 48 | 0.17 (-0.14, 0.48) | 50 | 0.35 (0.01, 0.69) | 50 | 1.3 (0.7, 2.2) | 50 | 1.5 (0.8, 2.7) |
|  | household soil | 72 | 0.12 (-0.14, 0.38) | 71 | 0.11 (-0.18, 0.39) | 71 | 0.9 (0.1, 2.5) | 71 | 0.7 (0.1, 1.9) |
|  | stored water | 80 | -0.28 (-0.66, 0.06) | 80 | -0.02 (-0.24, 0.21) | 80 | 1.8 (0.5, 6.3) |  |  |
|  | food surface | 79 | -0.08 (-0.59, 0.43) | 79 | 0.15 (-0.09, 0.39) |  |  |  |  |
| persons per latrine | latrine soil | 53 | 0.10 (-0.17, 0.38) | 55 | 0.41 (0.10, 0.72) | 55 | 1.9 (1.0, 3.5) | 55 | 2.6 (1.1, 5.5) |
|  | household soil | 83 | 0.27 (0.09, 0.46) | 82 | 0.20 (-0.02, 0.42) | 82 | 0.9 (0.3, 1.7) | 82 | 1.7 (0.8, 3.8) |
|  | stored water | 91 | 0.00 (-0.31, 0.31) | 91 | 0.04 (-0.15, 0.21) | 91 | 1.8 (0.5, 5.8) |  |  |
|  | food surface | 90 | -0.16 (-0.57, 0.23) | 89 | -0.10 (-0.32, 0.11) |  |  |  |  |
| yesterday mean temp (°C) | latrine soil | 54 | -0.21 (-0.46, 0.05) | 56 | -0.02 (-0.33, 0.29) | 56 | 0.6 (0.3, 1.1) | 56 | 0.9 (0.5, 1.6) |
|  | household soil | 85 | -0.04 (-0.27, 0.19) | 84 | 0.04 (-0.19, 0.26) | 84 | 0.6 (0.2, 1.1) | 84 | 0.5 (0.1, 1.1) |
|  | stored water | 91 | -0.08 (-0.44, 0.26) | 91 | -0.08 (-0.28, 0.12) | 91 | 1.0 (0.2, 2.8) |  |  |
|  | food surface | 90 | -0.09 (-0.56, 0.38) | 89 | -0.20 (-0.41, 0.02) |  |  |  |  |
| yesterday max temp (°C) | latrine soil | 54 | -0.22 (-0.50, 0.06) | 56 | 0.11 (-0.21, 0.43) | 56 | 0.5 (0.2, 0.8) | 56 | 0.8 (0.4, 1.4) |
|  | household soil | 85 | -0.18 (-0.40, 0.03) | 84 | -0.11 (-0.34, 0.12) | 84 | 0.8 (0.3, 1.5) | 84 | 0.3 (0.1, 0.7) |
|  | stored water | 91 | -0.36 (-0.68, -0.03) | 91 | -0.06 (-0.27, 0.16) | 91 | 1.1 (0.2, 3.1) |  |  |
|  | food surface | 90 | -0.07 (-0.53, 0.39) | 89 | 0.13 (-0.10, 0.35) |  |  |  |  |
| past week rain (cm) | latrine soil | 54 | 0.00 (-0.11, 0.11) | 56 | 0.03 (-0.10, 0.17) | 56 | 0.9 (0.6, 1.1) | 56 | 1.0 (0.8, 1.3) |
|  | household soil | 85 | -0.05 (-0.14, 0.04) | 84 | 0.01 (-0.08, 0.10) | 84 | 1.1 (0.7, 1.4) | 84 | 1.0 (0.6, 1.4) |
|  | stored water | 91 | -0.19 (-0.33, -0.05) | 91 | 0.00 (-0.08, 0.09) | 91 | 0.7 (0.2, 1.3) |  |  |
|  | food surface | 90 | -0.01 (-0.19, 0.18) | 89 | 0.08 (-0.01, 0.18) |  |  |  |  |
| past month rain (cm) | latrine soil | 54 | 0.07 (-0.01, 0.15) | 56 | -0.09 (-0.19, 0.00) | 56 | 1.0 (0.8, 1.1) | 56 | 1.1 (0.9, 1.3) |
|  | household soil | 85 | 0.01 (-0.05, 0.08) | 84 | 0.04 (-0.03, 0.11) | 84 | 1.0 (0.8, 1.3) | 84 | 1.2 (0.9, 1.7) |
|  | stored water | 91 | 0.04 (-0.07, 0.15) | 91 | 0.00 (-0.06, 0.06) | 91 | 0.7 (0.3, 1.1) |  |  |
|  | food surface | 90 | -0.01 (-0.16, 0.12) | 89 | 0.02 (-0.05, 0.09) |  |  |  |  |
| rainy days past week | latrine soil | 54 | 0.19 (-0.10, 0.48) | 56 | 0.05 (-0.33, 0.44) | 56 | 1.4 (0.7, 2.5) | 56 | 2.9 (1.2, 6.3) |
|  | household soil | 85 | 0.26 (0.02, 0.50) | 84 | 0.21 (-0.03, 0.47) | 84 | 0.9 (0.3, 2.0) | 84 | 1.5 (0.5, 3.9) |
|  | stored water | 91 | 0.43 (0.05, 0.81) | 91 | 0.02 (-0.22, 0.24) | 91 | 1.9 (0.5, 6.0) |  |  |
|  | food surface | 90 | 0.43 (-0.05, 0.92) | 89 | -0.14 (-0.40, 0.11) |  |  |  |  |
| rainy days past month | latrine soil | 54 | 0.02 (-0.12, 0.16) | 56 | 0.00 (-0.17, 0.16) | 56 | 1.2 (0.9, 1.6) | 56 | 1.4 (1.1, 2.0) |
|  | household soil | 85 | 0.12 (0.02, 0.23) | 84 | 0.13 (0.03, 0.24) | 84 | 1.1 (0.7, 1.7) | 84 | 1.8 (1.2, 2.9) |
|  | stored water | 91 | 0.26 (0.08, 0.44) | 91 | 0.01 (-0.09, 0.12) | 91 | 1.4 (0.7, 3.2) |  |  |
|  | food surface | 90 | 0.15 (-0.08, 0.39) | 89 | -0.23 (-0.32, -0.13) |  |  |  |  |
