# Supplementary material for: Human fecal contamination of water, soil, and surfaces in households sharing poor-quality sanitation facilities in Maputo, Mozambique
Source: Int J Hyg Environ Health. Author manuscript; Available in PMC 2020 May 1. (PMC7174141; doi:10.1016/j.ijheh.2020.113496)
Supplement: Supplementary file 4 [file NIHMS1570378-supplement-Table_S7.docx]

Table S7. Estimated associations between environmental sample characteristics and microbial indicators of fecal contamination from univariable, multilevel Bayesian regression using censored linear models for *E. coli* concentration responses and logistic models for human target detection responses

|  |  | **cEC** |  | **EC23S** |  | **HF183** |  | **any human** |  |
| --- | --- | --- | --- | --- | --- | --- | --- | --- | --- |
| **sample type** | **characteristic** | **n** | **Δ log**_**10**_ **cfu (95% CI)** | **n** | **Δ log**_**10**_ **gc (95% CI)** | **n** | **OR (95% CI)** | **n** | **OR (95% CI)** |
| latrine soil | reduced sun exposure | 49 | 0.23 (-0.43, 0.91) | 49 | 0.29 (-0.48, 1.05) | 49 | 3.52 (0.70, 11.71) | 49 | 1.67 (0.33, 4.99) |
| latrine soil | visibly wet | 49 | 0.67 (0.09, 1.26) | 49 | 1.08 (0.45, 1.74) | 49 | 38.53 (2.98, 238.23) | 49 | 6.61 (1.54, 20.18) |
| household soil | reduced sun exposure | 85 | 0.20 (-0.31, 0.72) | 84 | 0.04 (-0.46, 0.54) | 84 | 0.59 (0.16, 1.66) | 84 | 0.75 (0.21, 1.99) |
| household soil | visibly wet | 83 | 0.45 (0.07, 0.85) | 82 | 0.27 (-0.13, 0.68) | 82 | 1.08 (0.36, 2.61) | 82 | 1.17 (0.46, 2.53) |
| stored water | extracted by dipping | 89 | 0.37 (-0.29, 1.05) | 89 | -0.18 (-0.59, 0.21) | 89 | 5.27 (0.88, 19.37) | 88 | 4.85 (0.81, 19.69) |
| stored water | plastic container | 90 | -0.50 (-1.75, 0.69) | 90 | -0.38 (-1.09, 0.35) | 90 | 0.31 (0.04, 1.20) | 89 | 0.30 (0.03, 1.14) |
| stored water | uncovered container | 89 | -0.10 (-0.77, 0.57) | 89 | 0.26 (-0.11, 0.64) | 89 | 2.63 (0.74, 6.71) | 88 | 2.81 (0.69, 7.44) |
| stored water | wide mouth container | 89 | 0.38 (-0.32, 1.06) | 89 | -0.19 (-0.58, 0.20) | 89 | 4.72 (0.82, 18.95) | 88 | 4.27 (0.75, 15.53) |
| food surface | bowl surface | 90 | 0.05 (-1.59, 1.56) | 89 | -1.11 (-1.76, -0.47) |  |  |  |  |
| food surface | plastic surface | 90 | 0.97 (-0.32, 2.22) | 89 | -0.82 (-1.37, -0.25) |  |  |  |  |
